# JCoDA: a tool for detecting evolutionary selection

**DOI:** 10.1186/1471-2105-11-284

**Published:** 2010-05-27

**Authors:** Steven N Steinway, Ruth Dannenfelser, Christopher D Laucius, James E Hayes, Sudhir Nayak

**Affiliations:** 1 Department of Biology, The College of New Jersey, 2000 Pennington Road, Ewing, NJ 08628, USA; 2 Weill Cornell Graduate School of Medical Sciences, 1300 York Ave, Box 65, New York, NY 10065, USA

## Abstract

**Background:**

The incorporation of annotated sequence information from multiple related species in commonly used databases (Ensembl, Flybase, Saccharomyces Genome Database, Wormbase, etc.) has increased dramatically over the last few years. This influx of information has provided a considerable amount of raw material for evaluation of evolutionary relationships. To aid in the process, we have developed JCoDA (Java Codon Delimited Alignment) as a simple-to-use visualization tool for the detection of site specific and regional positive/negative evolutionary selection amongst homologous coding sequences.

**Results:**

JCoDA accepts user-inputted unaligned or pre-aligned coding sequences, performs a codon-delimited alignment using ClustalW, and determines the dN/dS calculations using PAML (Phylogenetic Analysis Using Maximum Likelihood, yn00 and codeml) in order to identify regions and sites under evolutionary selection. The JCoDA package includes a graphical interface for Phylip (Phylogeny Inference Package) to generate phylogenetic trees, manages formatting of all required file types, and streamlines passage of information between underlying programs. The raw data are output to user configurable graphs with sliding window options for straightforward visualization of pairwise or gene family comparisons. Additionally, codon-delimited alignments are output in a variety of common formats and all dN/dS calculations can be output in comma-separated value (CSV) format for downstream analysis. To illustrate the types of analyses that are facilitated by JCoDA, we have taken advantage of the well studied sex determination pathway in nematodes as well as the extensive sequence information available to identify genes under positive selection, examples of regional positive selection, and differences in selection based on the role of genes in the sex determination pathway.

**Conclusions:**

JCoDA is a configurable, open source, user-friendly visualization tool for performing evolutionary analysis on homologous coding sequences. JCoDA can be used to rapidly screen for genes and regions of genes under selection using PAML. It can be freely downloaded at http://www.tcnj.edu/~nayaklab/jcoda.

## Background

The first step in the assessment of evolutionary relationships between related sequences is the generation of pairwise or multiple sequence alignments (MSAs). Over the last two decades several algorithms have been developed to generate rapid yet accurate sequence alignments for subsequent analysis [1]. A commonly used program, ClustalW, generates MSAs of DNA or amino acids by constructing a branched guide tree from pairwise alignments [2]. More recent progressive alignment methods, such as T-COFFEE, have improved the accuracy of ClustalW by combining information from local and global alignments [3]. Other methods of sequence alignment, such as iterative, all-against-all, and hybrid approaches have also been shown to improve the accuracy of ClustalW, although some necessitate significant increases in computational power [4-7]. Regardless of the method, when DNA is aligned it is done so in a manner that arranges sequences to minimize gaps and mismatches to achieve a maximal score based on sequence identity and similarity.

**Figure 1:**
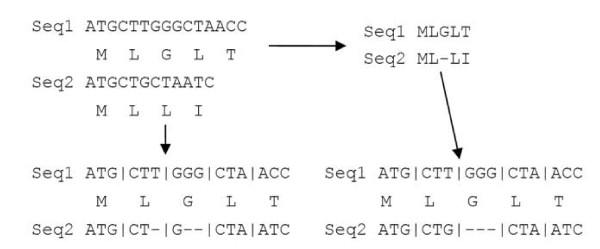
**Codon delimited multiple sequence alignment**. Two unaligned cDNA sequences with translated peptides (top-left). Peptide sequences are aligned (top-right) and cDNA codons are matched to their corresponding amino acids to create the codon-delimited alignment (bottom-right). Aligning the sequences by cDNA rather than by peptide results in partitioning of codons (bottom-left).

Given that codon triplets are considered the unit of coding sequence evolution, DNA alignments that do not constrain codons are likely to misrepresent the encoded information [8]. For example, to meet the optimality criteria used in the alignment of DNA sequences, single gaps are frequently inserted and thus distort the reading frame (Figure 1, left). Essentially, for coding sequences, an "optimal" alignment of DNA may ignore the rules that govern its translation into protein. As a result, the evolutionary constraints placed on the protein product are lost in the analysis. A straightforward solution to the problem would be to perform a codon-based alignment that does not allow the partition of codons (Figure 1, right). The codon-based alignment can then be used to detect adaptive molecular evolution or purifying selection by estimating the number of non-synonymous and synonymous substitutions (dN/dS). In general, the aligned sequences are screened for dN/dS ratios of >1 (adaptive) or dN/dS ratios of < 1 (purifying).

There are currently several informatics tools freely available that calculate dN/dS ratios to measure evolutionary selection or generate codon-delimited alignments. The online program OCPAT is able to generate codon-delimited of alignments from human gene IDs and their putative orthologs from other vertebrates; however, OCPAT is not able to calculate dN/dS scores [9]. Most of the programs that calculate dN/dS require a codon-delimited alignment. For example, the programs SNAP (Synonymous Non-synonymous Analysis Program) and WINA (Window Analysis) employ user provided alignments to calculate substitution rates, where WINA also allows for the use of sliding window analysis [10-12]. SWAKK (Sliding Window Analysis of Ka and Ks) uses pairwise sequence alignments, sliding windows, and structural alignment to identify regions of positive selection [13]. DNaSP (DNA Sequence Polymorphism) allows for the detection of diversifying selection by measuring DNA polymorphisms and also uses sliding window analysis [14].

In an effort to simplify the process, PAL2NAL (v12) takes pre-aligned protein and the corresponding unaligned coding DNA to generate codon-delimited alignments. While useful, PAL2NAL is constrained to pairwise dN/dS analysis, does not include graphing, and does not allow the user to edit the codeml control file [15]. The popular program PAML (Phylogenetic Analysis by Maximum Likelihood) is able to perform both pairwise and site-based dN/dS calculations. However, it does not have a GUI (graphical user interface) and visualization of the output data can be cumbersome. In addition, it requires the input of pre-aligned sequences [8]. More recently, IDEA (Interactive Display For Evolutionary Analyses) has implemented a GUI for both PAML and Phylip (Phylogeny Inference Package) and is amenable for high throughput genome-wide analysis [4]. While IDEA is a powerful tool, it does not run on the Windows operating system, requires the separate installation of several support programs, uses multiple languages, generates graphs that are difficult to configure, and also requires a codon-delimited alignment.

We have designed JCoDA to be an easy-to-use tool that integrates several common functions required for the detection of evolutionary selection in coding sequences. Specifically, the steps that are now controlled from the JCoDA interface: generation of codon-delimited alignments, generation of phylogenetic trees, estimation of site and regional dN/dS scores under multiple models of substitution, and the generation of user configurable graphical output. JCoDA only requires unaligned coding sequences (CDS) in FASTA format and it takes advantage of the freely available BioJava framework [16], ClustalW [17], Phylip [18], and PAML [8] to identify positive/negative evolutionary selection. The visualization options in JCoDA include a variety of common alignment formats, and user configurable scalable graphs with sliding window options for pairwise or gene family comparisons. To demonstrate the types of analyses facilitated by JCoDA, we have performed an analysis of 25 sex determination genes in nematodes and identified genes under positive selection, regional positive selection, and differences in selective pressure likely due to functional constraints.

## Implementation

JCoDA was designed to assist in performing common operations associated with evolutionary analysis. It coordinates the passage of information from the initial alignments (ClustalW version 2.0) to the calculation of dN/dS (yn00 or codeml, version 4.1) to the visualization of output. For site-based analysis of selection we have packaged JCoDA with a graphical interface for Phylip (version 3.6) that allows for the generation of phylogenetic trees. JCoDA is written entirely in Java, which allows for the addition of supplementary modules that offer additional functionality. To allow for easy installation, JCoDA is packaged with all of the required components (ClustalW, Phylip, and PAML) and can be installed on any computer with a Windows operating system (or virtual machine) and Java Runtime Environment 6 (JRE 6) (if Java 1.6 has been installed). For ease of operation, installation of Java Developer Kit 6 bundled with NetBeans (JDK, which includes JRE 6) is recommended http://java.sun.com/javase/downloads/index.jsp.

JCoDA provides two options for calculation of dN/dS scores: regional pairwise calculation via sliding window or a site-specific calculation. Pairwise dN/dS performs the calculation between any user-selected sequences from a list of all possible comparisons presented in the GUI. For sliding window pairwise calculations, the size of the window, jump, and substitution models are configurable via drop down menus. Once the selected sequence comparisons are submitted, JCoDA parses through them by window, converts them to Phylip format, and feeds each window to PAML (yn00) suite to calculate dN/dS. JCoDA does this iteratively over all windows until the end of the selected sequence pair. The potential benefit of the sliding window option is that it can be performed very quickly and it is able to extract information about regional selection. However, it is important to note that methods that incorporate sliding windows have been demonstrated to be prone to artifact arising due to resampling and illustrate the importance of incorporating site-based methods and the likelihood ratio test in sequence analysis [19].

JCoDA takes advantage of the codeml executable included in the PAML package to implement site-specific dN/dS calculation. Similar to pairwise comparisons, JCoDA converts all inputted sequences to Phylip format and feeds them to codeml. In order for a site-based dN/dS calculation to be performed, the user must provide a tree file and set its path for use in the GUI. To allow for this operation, the JCoDA package includes a Java based graphical interface for the Phylip package [18], http://evolution.genetics.washington.edu/phylip.html). The Phylip Graphical Interface (PGI) allows for the generation of trees using neighbor-joining, parsimony, or maximum likelihood based methods of either DNA or protein. Regardless of the source of the tree file, JCoDA will accept any tree in Phylip format.

JCoDA implements M7 (fit to a beta distribution, dN/dS > 1 disallowed) -vs- M8 (fit to a beta distribution, dN/dS > 1 allowed) and M1a (nearly neutral) -vs- M2a (positive selection) in conjunction with the likelihood ratio test to check for evidence of positive selection [20]. To maintain JCoDA's flexibility, the user is given the option to access the complete codeml control file as a selectable advanced options tab to vary other parameters.

## Results and Discussion

### Input requirements and alignments

The JCoDA user interface, input requirements, and installation have been designed to be easier to use while retaining the underlying power of the codeml and yn00 programs from the PAML package (Additional File 1, Additional File 2, Additional File 3, Additional File 4 and Additional File 5). For example, we have simplified the input requirements to the coding sequences (CDS) in FASTA format. There were two primary reasons for this streamlined approach. First, CDS can be readily batch retrieved from NCBI or organism specific databases such as Wormbase (WormMart) [20]. Second, the risk of mismatch between DNA and protein sequences is eliminated by directly translating the CDS input by the user.

For unaligned CDS, the sequences are translated by the BioJava framework [16] and the proteins are passed to ClustalW for alignment. JCoDA generates a codon-delimited alignment using the protein alignment from ClustalW as a guide to prevent the interruption of codon triplets, (Figure 1). JCoDA also accepts pre-aligned protein sequences in FASTA format paired with corresponding unaligned CDS in the same format to allow for the use of sequences aligned in another program or modified by hand. When provided with prealigned protein sequences and corresponding CDS, JCoDA simply circumvents ClustalW and performs the codon-delimited alignment directly.

### Visualization and output

**Figure 2:**
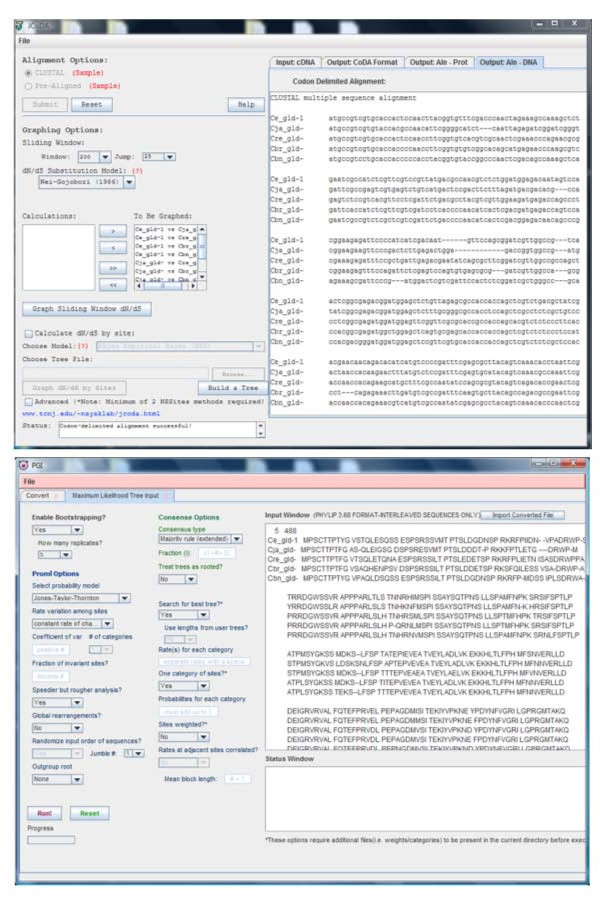
**JCoDA interface**. **A) **The JCoDA interface accepts cDNA in FASTA format and JCoDA manages the generation of codon delimited alignments, pairwise or site-based dN/dS calculation through PAML, and graphing of either output (top). The codon delimited alignment tab is shown. Common options are displayed on the main interface and the file menu contains export options. Outputs, such as raw data, alignments, and graphs are displayed in tabbed panes for easy navigation. Graphs for multiple models are retained in the tabbed interface for comparison. For pairwise dN/dS the user the selects sequences from a list using add/remove buttons. The advanced options check box provides direct access to the codeml control file as a tabbed pane. For site-based analysis JCoDA defaults to implement models M7 (null, neutral) -vs- M8 (alternate, selection) in codeml. The user can set the path for their own tree file or use the PHYLIP graphical interface (PGI) to generate phylogenetic a tree (bottom).

**Figure 3:**
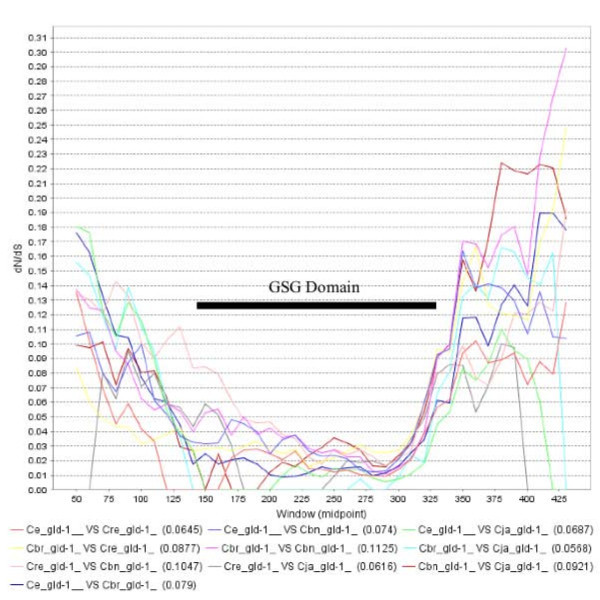
**Sample of JCoDA output using sliding window analysis of pairwise *gld-1 *dN/dS**. Purifying selection dominates the RNA binding GSG domain (amino acids 141-328, *Ce_gld-1*). All pairwise comparisons suggested relaxed puryfing selection at the N (left) and C-terminal (right) ends. Black bar = GSG domain amino acids 141-328 relative to *Ce_gld-1*. *Ce = C. elegans, Cbr = C. briggsae, Cre = C. remanei, Cbn = C. brenneri*, and *Cja = C. japonica*. All pairwise comparisons were performed using a window 100 and jump of 10. The graph was saved directly from JCoDA and GSG and bar were added in Microsoft Word.

**Figure 4:**
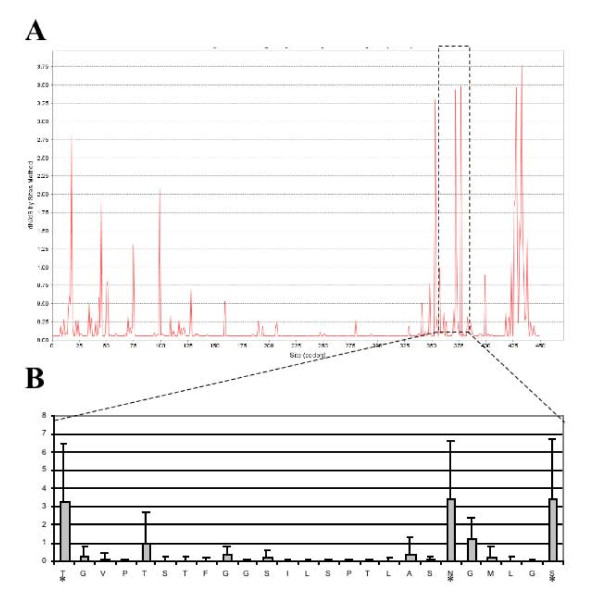
**Results for *gld-1 *family site-based analysis**. **A) **Graph of M8 (BEB, Bayes Empirical Bayes) from codeml. Peaks represent sites where relaxed purifying or positive selection was detected. Similar to Figure 3, extensive purifying selection dominates the RNA binding GSG domain (amino acids 141-328, *Ce_gld-1*). **B) **Higher magnification image of dashed region from part A with Excel processed CSV output. Amino acids 354 to 378 are on the x-axis, dN/dS by site on y-axis, and error bars from codeml. "*" indicates residues with dN/dS >1 (p > 0.5). Graphical output from A is directly from JCoDA. The dashed box was added in Microsoft Word.

Navigation in JCoDA has been designed around the use of a tabbed GUI that allows for shuffling between graphs and sequence alignments (Figure 2). We have implemented JFreeChart http://www.jfree.org to generate robust graphs of dN/dS scores by both sliding window and site-specific methods. The graphs are extensively customizable, dynamically scaled, and can be saved as PNG (Portable Network Graphics) files (Figure 3 and 4A). The user can export protein and DNA alignment files in ClustalW, Phylip (v3.2 and v4), and hybrid protein/DNA codon-delimited formats using the file menu. The dN/dS scores from all models selected, including p values, can be exported as common separated values (CSV) files allowing for further analysis in database, spreadsheet, and graphing programs (Figure 4B).

### Benchmarking

The use of JCoDA interface does not add significantly to the time need required to run ClustalW, Phylip, or PAML. Any time costs incurred are more than ameliorated by the integration of multiple tasks. For example, the user does not have to reformat sequences to shuttle them from one program to another or use an additional program to obtain graphical representations of the data. To test the ability of JCoDA to recover signatures of directional selection, we used multiple datasets including TRIM5α (hominids + OWMs) [21], *nef *gene of HIV-1 (pairwise and ML) [22], lysozyme [23], and a subset of HIV1 protease and reverse transcriptase sequences from the HIV positive selection mutation database [24]. We have included an analysis of the sex determination genes in nematodes to illustrate the utility of JCoDA in identification of directional selection.

### Evolutionary selection in the nematode sex determination pathway

The extreme divergence in sex determination pathway components has made their analysis problematic using comparison of distantly related species (e.g. *C. elegans *-vs- *D. melanogaster*) The recent availability of sequence information from nematodes related to *Caenorhabditis elegans *(*C. elegans*) has provided the raw material for the analysis of sex determination pathway components where sequence divergence has not erased all evidence of directional selection. We used the JCoDA interface to perform a screen for directional selection using all known sex determination pathway components in nematodes closely related to *C. elegans*. We were able to identify genes that have been previously shown (or suspected) to be under positive selection. Furthermore, we identified differences in selection between genes based on their function in the sex determination cascade (Table 1).

**Table 1:** Analysis of sex determination pathway genes

Gene	Pathway	dN/dS	M7-vs-M8	Proportion of sites with dN/dS>1
*fem-1*	B	0.13	0.86	0.7
*fem-2*	B	0.12	3.49	2.6
*fem-3*	B	0.27	0.99	3.8
*her-1*	B	0.19	0.09	0.6
*laf-1*	B	0.04	2.34	1.1
*tra-1*	B	0.25	**7.93**	6.0
*tra-2*	B	0.29	1.60	0.0

*fox-1*	S	0.06	0.02	0.7
*sdc-1*	S	0.24	0.72	0.0
*sdc-2*	S	0.35	3.30	0.0
*sdc-3*	S	0.45	**7.21**	7.8
*sea-1*	S	0.57	**11.44**	52.5
*sea-2*	S	0.32	**20.11**	21.7
*sel-10*	S	0.06	**8.21**	1.7
*sex-1*	S	0.22	**12.47**	1.6
*xol-1*	S	0.32	2.66	0.0

fbf-1/2*	G	0.15	3.93	1.3
*fog-1*	G	0.17	**12.30**	4.5
fog-2*	G	0.51	**48.23**	19.2
*fog-3*	G	0.18	0.67	2.0
*gld-1*	G	0.07	**7.17**	1.6
*gld-3*	G	0.35	**36.55**	10.9
*mog-1*	G	0.02	4.87	0.4
*mog-6*	G	0.04	1.09	0.2
*nos-3*	G	0.39	**50.41**	22.5

Genes are sorted based on functions in germ line and somatic sex determination (B), somatic sex determination (dosage compensation) (S), or germ line sex determination (G). All sex determination gene orthologs used were identified by reciprocal best-hit or conserved synteny (Wormbase). Only genes with large indels and obvious mispredictions that could be resolved were eliminated. dN/dS = average pairwise score for the gene family (Nei & Gojobori, 1986). Models compared for likelihood ratio test (LRT) were M7(dN/dS>1 disallowed, null) -vs- M8(dN/dS>1 allowed, alternate), where bold indicates number higher than critical value (Df = 2, χ^2^_l _= 5.9915, p = 0.05) and rejection of the null. Portion of sites with dN/dS>1 was calculated as a ratio of selected sites with a probability higher than 0.5 of dN/dS>1 to total sites. Tree files were based on the ClustalW alignments using the neighbor-joining method (Phylip). See associated FASTA files for ID numbers and species used. (*) = species specific gene expansion or contains species specific duplications.

Relative to the genome wide *C. elegans/C. briggsae *dN/dS ratio of 0.06 [25], the majority of sex determination genes show elevated levels of non-synonymous substitutions in pairwise comparisons (Table 1, dN/dS, 0.06 -vs- 0.23). Considerable variability within each category was present but there are no significant differences in average pairwise dN/dS scores between the categories. Interestingly, site-based analysis reveals evidence for positive selection in five of nine genes involved exclusively in germ line sex determination and five of nine genes involved in the specification of somatic sex (dosage compensation). In contrast, positive selection was only detected in one gene of seven genes involved in both germ line and somatic pathways (Table 1, grey). Evidence of fewer genes that function in multiple pathways with signatures of positive selection likely reflects resistance to change based on additional function constraints.

Positive selection can be difficult to detect when high levels of sequence divergence are present and dS is essentially saturated [26]. Haag and Ackerman (2005) [27] measured nucleotide diversity using sliding windows to detect patches of diversifying selection in *C. remanei fem-3 *(**fem**inization in XX and XO). We processed the same dataset with JCoDA and confirmed the presence of positive selection clustered between amino acids 339 and 408 (relative to AY142113), a highly polymorphic region of the protein (Table 2). Also consistent with their data, the *tra-2 *region used did not show evidence of positive selection, although the dN/dS ratio was elevated (Table 2, dN/dS, 0.37). As additional intraspecies sequence information is collected, it is likely that the capability to detect positive selection will be significantly enhanced.

**Table 2:** Analysis of *C. remanei fem-3 *and *tra-2 *genes

Gene	Pathway	dN/dS	M7-vs-M8	Proportion of sites with dN/dS>1
*fem-3*	B	0.23	**10.42**	2.3
*tra-2*	B	0.37	1.23	0.7

The *fem-3 *and *tra-2 *intraspecies data from Haag and Ackerman (2005) were analyzed using both pairwise and site-based methods. Using M7 -vs- M8 models LRT rejects the null for *fem-3 *(Df = 2, χ^2^_l _= 9.2103, P < 0.01, bold) but not for *tra-2*. B = involved in somatic and germ line sex determination. dN/dS = average pairwise. Proportion of sites with dN/dS>1 = ratio of selected sites with a probability higher than 0.5 of dN/dS>1 to total sites.

Examples of the graphical output generated by JCoDA are shown in Figures 3 and 4. The GLD-1 (defective in germ line development) RNA binding protein governs the translation of numerous mRNA targets including functioning with FOG-2 in the promotion of spermatogenesis in the hermaphrodite [26,28,29]. As expected, pairwise sliding window analysis reveals that extensive purifying selection dominates regions with homology to the RNA binding GSG (GRP33/Sam68/GLD-1) domain (Figure 3, GSG domain). Interestingly, elevated levels of dN/dS were detected at both the N and C-terminal ends relative to the average pairwise dN/dS for *gld-1 *(0.07). Using site-based analysis, we confirmed the presence of residues under positive selection (Figure 4B). Curiously, even though GLD-1 orthologs in *C. elegans *and *C. briggsae *share significant amino acid homology (80% identity, 90% similarity) and have a dN/dS ratio consistent with purifying selection, they have opposite functions in sex determination [30]. We speculate that species-specific functions could be explained by at least two mechanisms. First, the extensive conservation in the KH-domain between *C. elegans *and *C. briggsae *suggests that at least some of the species differences in GLD-1 function can be explained by a change in mRNA targets. Second, based on the elevated non-synonymous substations at the N and C-terminal ends, we can infer that some changes in GLD-1 function could result from species-specific interactions or regulation.

## Conclusions

An important part of the analysis of homologous coding sequences is the characterization of evolutionary selection by comparing the rates of synonymous and non-synonymous substitutions. The primary issues with these types of analyses are the difficulty in generating codon-delimited alignments, shuttling between programs, and the complexity in configuring programs that are designed to detect positive selection. JCoDA provides a simple platform that integrates several common functions associated with evolutionary analysis of coding sequences and the detection of positive/negative selection. JCoDA is a modular tool built using the BioJava framework, ClustalW, Phylip, and PAML that allows for the rapid assessment and visualization of the pairwise and site-based selection pressure on coding sequences. Using JCoDA we were able to identify multiple sex determination pathway genes with evidence of positive selection based on functional constraints. The JCoDA executable, source code, and tutorial are freely available at http://www.tcnj.edu/~nayaklab/jcoda (Additional File 6).

## Availability and requirements

Project name: JCoDA: A Tool for Detecting Evolutionary Selection

Project home page: http://www.tcnj.edu/~nayaklab/jcoda

Operating system(s): Windows

Programming language: Java

Other requirements: Java Runtime Environment 6.0 (JRE 6) or Java Developer Kit 6 (JDK 6, includes JRE)

License: GPL GNU version 3 for JCoDA or PGI. Please do not violate the copyright or terms of use for ClustalW, Phylip, PAML, and JFreeChart.

Any restrictions to use by non-academics: JCoDA and PGI are provided free for academic use only. Please be aware of the copyright or terms of use for ClustalW, Phylip, PAML, and JFreeChart.

## Authors' contributions

RD: Designed and implemented the Phylip graphical interface, JH: Contributed to the initial development of JCoDA, CL: Designed the JCoDA interface, generated output formats, and implemented integration with ClustalW, SS: Integrated PAML functionality, implemented graphing, and performed sex determination gene analysis, SN: Initiated development of JCoDA, contributed to the analysis of sex determination genes, and coordinated the project. All authors contributed the writing of the manuscript and have approved its final version.

## Supplementary Material

Additional file 1 Zipped archive that contains the JCoDA/PGI tutorial (tutorial.pdf ), readme files (JCoDA readme.txt and PGI readme.txt), and video guides (JCoDA videos guide.txt and Common problems video guide.txt). Click here for file

Additional file 2 **Zipped archive that contains the JCoDA/PGI installation and configuration video (FAQ - Installing and configuring -long-.swf).** Not required for all installations. Click here for file

Additional file 3 Zipped archive that contains videos of checking Java version (FAQ Checking your version of Java -audio-.swf) and a sample of sliding window analysis. Click here for file

Additional file 4 Zipped archive that contains videos documenting common problems with JCoDA and PGI. Click here for file

Additional file 5 Zipped archive that contains videos documenting partial functionally with Mac OS X 10.6. Click here for file

Additional file 6 Zipped archive that contains all JCoDA source code and executable jar files. Click here for file
