# Supplementary material for: JCoDA: a tool for detecting evolutionary selection
Source: BMC Bioinformatics. 2010 May 27;11:284. doi: 10.1186/1471-2105-11-284 (PMC2887424; doi:10.1186/1471-2105-11-284)
Supplement: Additional file 1 — Zipped archive that contains the JCoDA/PGI tutorial (tutorial.pdf ), readme files (JCoDA readme.txt and PGI readme.txt), and video guides (JCoDA videos guide.txt and Common problems video guide.txt). [file 1471-2105-11-284-S1.ZIP › Additional File 1 - Readme_tutorial_video_guide/tutorial.pdf]

JCoDA and PGI Tutorial
Version 1.0
Date 03/16/2010

	Page
**1.1 Guidelines**	**2**
Requirements	
JCoDA package	
Input file formats	
License	
**1.2 Java Installation**	**3-4**
**Not required in all cases**	
**2.1 dN/dS calculation using sliding window analysis**	**5-11**
**3.1 dN/dS calculation using site-based methods**	**12-14**
**3.2 dN/dS advanced options**	**15**
**4.1 Generating trees using the Phylip Graphical Interface (PGI)**	**16-17**
**4.2 Exporting sequences from JCoDA to PGI**	**18-19**
**5.1 Troubleshooting and FAQ**	**20-21**
**6.1 References**	**22**

1.1 Guidelines

JCoDA (Java based codon-delimited alignment) uses ClustalW[1], Phylip[2], and PAML[3] to perform codon-delimited alignments and calculate dN/dS either by sliding windows or by site based methods. JCoDA includes PGI (Phylip Graphical Interface), a Java based graphical user interface for Phylip that works with JCoDA to allow for some PAML operations. PGI can also function as a standalone program for the generation of phylogenetic trees. This guide includes the basic operating instructions for JCoDA and PGI and is not intended to be a tutorial on how to use ClustalW, Phylip, or PAML. Before using JCoDA, users should be familiar with the underlying assumptions and limitations of the programs that are integrated by the interface.

Requirements

JCoDA (and PGI) will run on any Windows machine or Windows virtual machine with Java Runtime Environment 6 (JRE) (http://java.sun.com/javase/downloads/index.jsp). We recommend installing Java Developer Kit 6 or higher (JDK, which includes JRE) bundled with NetBeans 6.8 to allow for easy modifications to the user interface. Both JRE and the JDK/NetBeans bundle are freely available from Sun Microsystems. JCoDA has been tested natively on Windows XP, Vista, and 7 and through VMware Fusion 3 (http://www.vmware.com/), Parallels 5 (http://www.parallels.com/), and VirtualBox 3.1.2 (http://www.virtualbox.org/) on OS X 10.5.8. JCoDA is fully functional through virtual machines; however, performance can be compromised when using site-based methods for calculating dN/dS and generation of phylogenetic trees using maximum likelihood estimation.

JCoDA Package

JCoDA package comes as a zipped archive complete with all the programs required to run (provided JRE has been installed, see *Requirements*). Simply unzip the archive and JCoDA and PGI are in the main directory as clickable (executable) jar files.

Input file formats

JCoDA accepts CDS (coding sequence) sequence in FASTA format or as paired pre-aligned protein and unaligned CDS sequences in FASTA format. CDS sequences are generally defined as the sequence of nucleotides that correspond to the sequence of amino acids in a protein from the start codon to the stop codon; however, partial cDNA sequences can also be used and will be processed the same way. It is important to note, *sequence names are limited to a maximum or eight characters.* For sequence with names longer than eight characters, the first eight characters of each sequence must be unique. Example input in FASTA format (partial sequences from NCBI):

```
>GI10457 gi|195112587|ref|XM_002000818.1| Drosophila mojavensis
ATGAGTGTCTGTGAGAACAAGACCGTTGTGCAACAGCAATTGCAACAACAGGCCGCCGCTGCCGTTGCGG
>GJ23144 gi|195390286|ref|XM_002053764.1| Drosophila virilis
ATGAGTGTATGTGAGAACAAGACCGTTGTGCAACAGCAGTTGCAACAACAGGCCGCCGCTGCCGTTGCGG
>GK14241 gi|195453217|ref|XM_002073655.1| Drosophila willistoni
ATGAGTGTTTGTGAGAAGAACAACGTTGTGCAACATCAATTGCAACAGGCTGCCGCAGTTGCTGCAGCCG
```

Examples of CDS files for analysis are included in the sample data folder. To follow along with the tutorial in the ensuing text use the “gld-1 CDS” file included in the sample data folder.

License

JCoDA and PGI are provided as free software: you can redistribute it and/or modify it under the terms of the GNU General Public License as published by the Free Software Foundation (version 3).

1.2 Java Installation (not required in all cases)

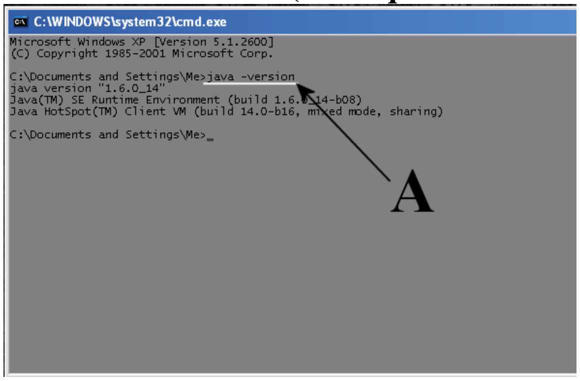

- After JDK or JRE+Java1.6 have been installed, from the command prompt type "java –version" (A). The text below should indicate that you have successfully installed Java 1.6 and JRE 1.6.
- Depending on your version of Windows the procedure below may or may not be required.

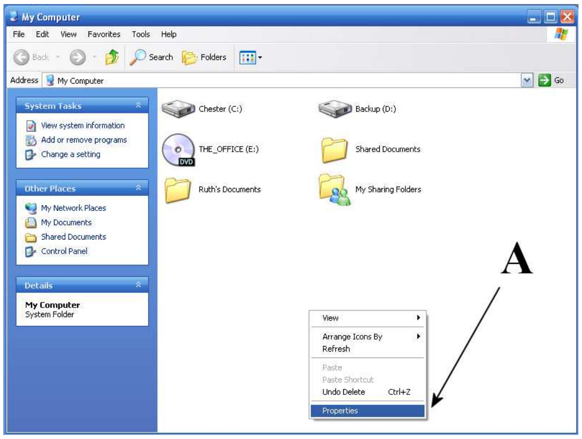

- Navigate to "My Computer" and then right-click and select properties form the menu (A)

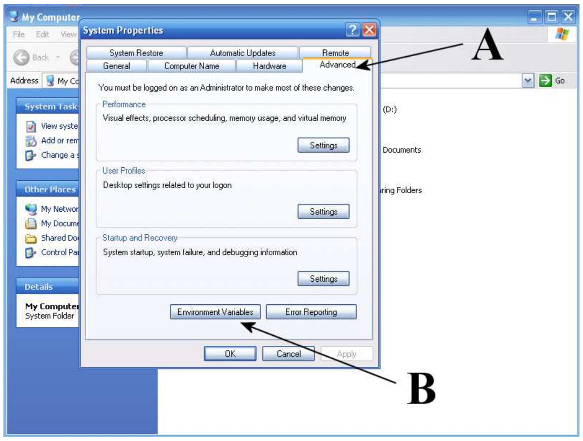

- Under "System Properties" navigate to the "Advanced" tab (A)
- Click "Environment Variables" (B)

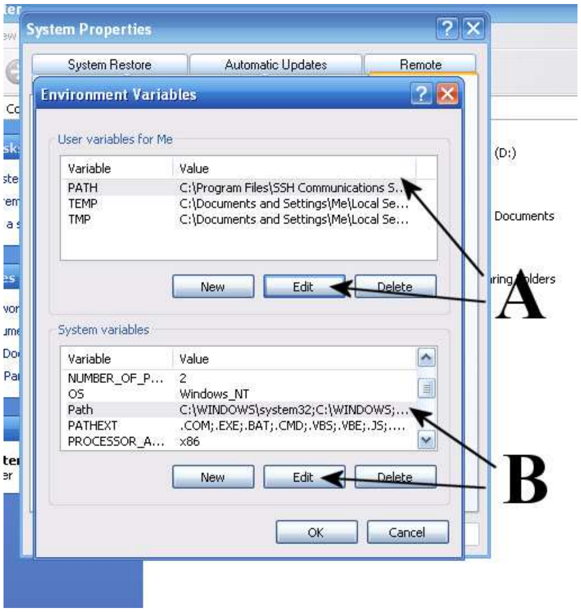

- Select "Path" under the "User variables" box and click "Edit" (A). *Note:You will need to repeat this procedure for the region indicated by "B".*

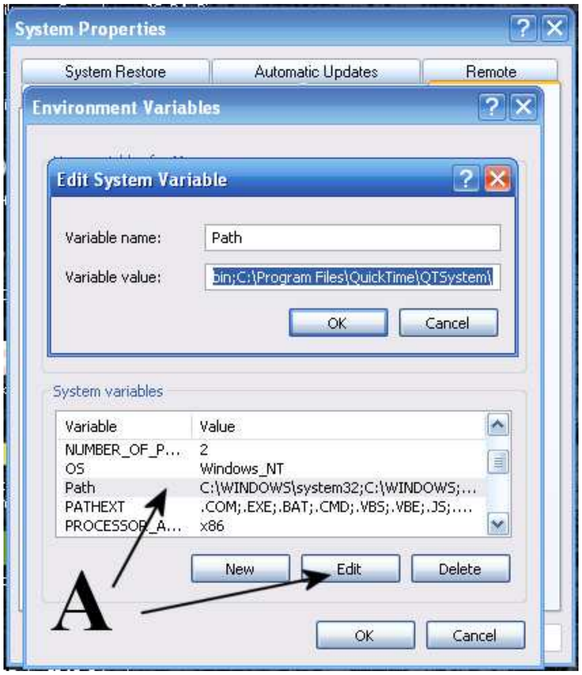

- In the window that appears add the path of your Java bin folder (e.g. C:\Program Files\Java1.6\JRE\bin;) to the end of the "variable value" line. Your path will differ depending on where Java has been installed. Don't forget to repeat this step for the "System variables" box indicated by (A).

2.1 dN/dS calculation using sliding window analysis

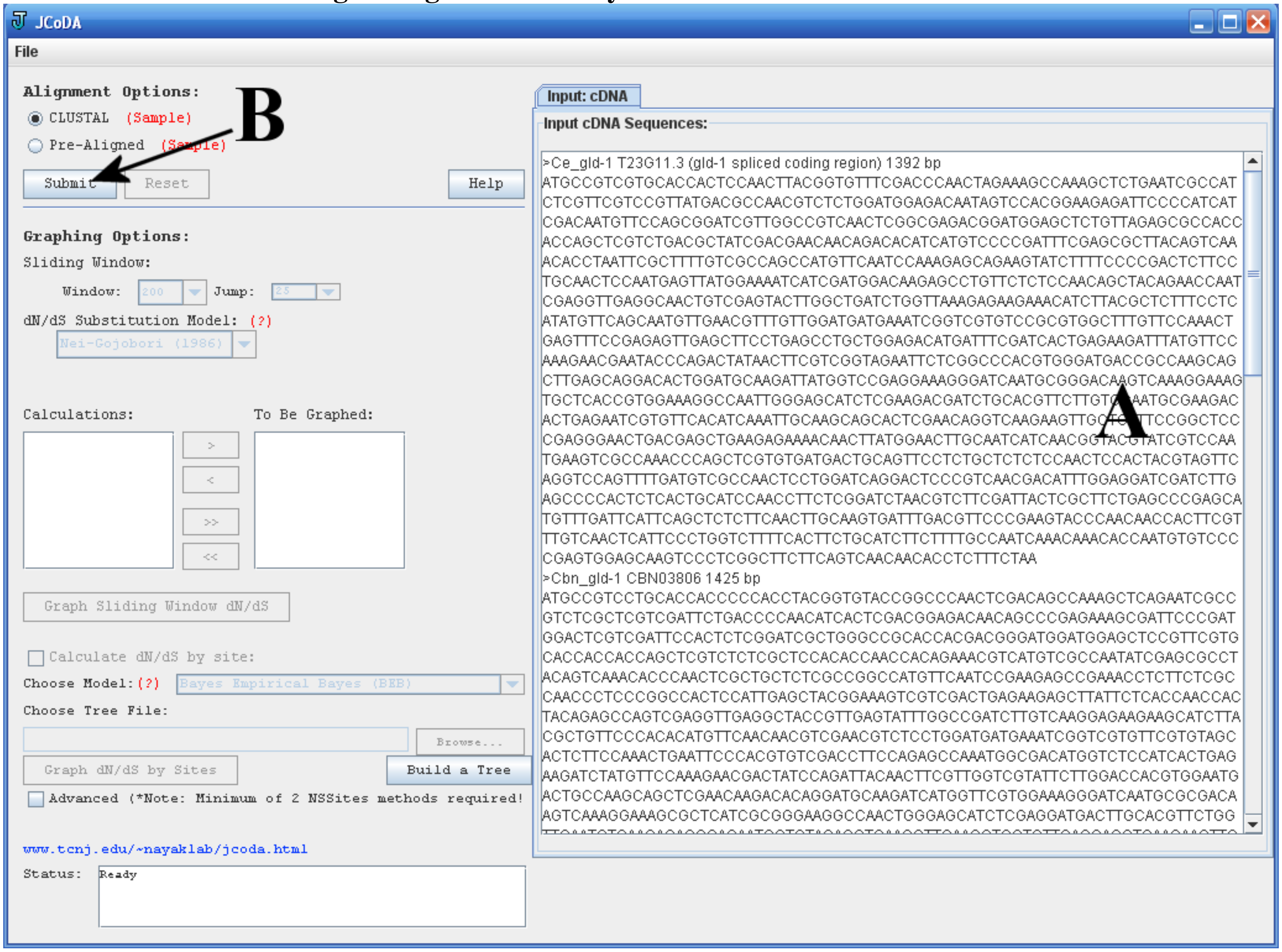

- Paste cDNA sequence into window (gld-1 CDS from sample data are shown) (A)
- If you are running JCoDA through a virtual machine on a Mac then DO NOT cut and paste from SimpleText – use the Windows Notepad program to avoid problems with line endings
- Click submit button (B)

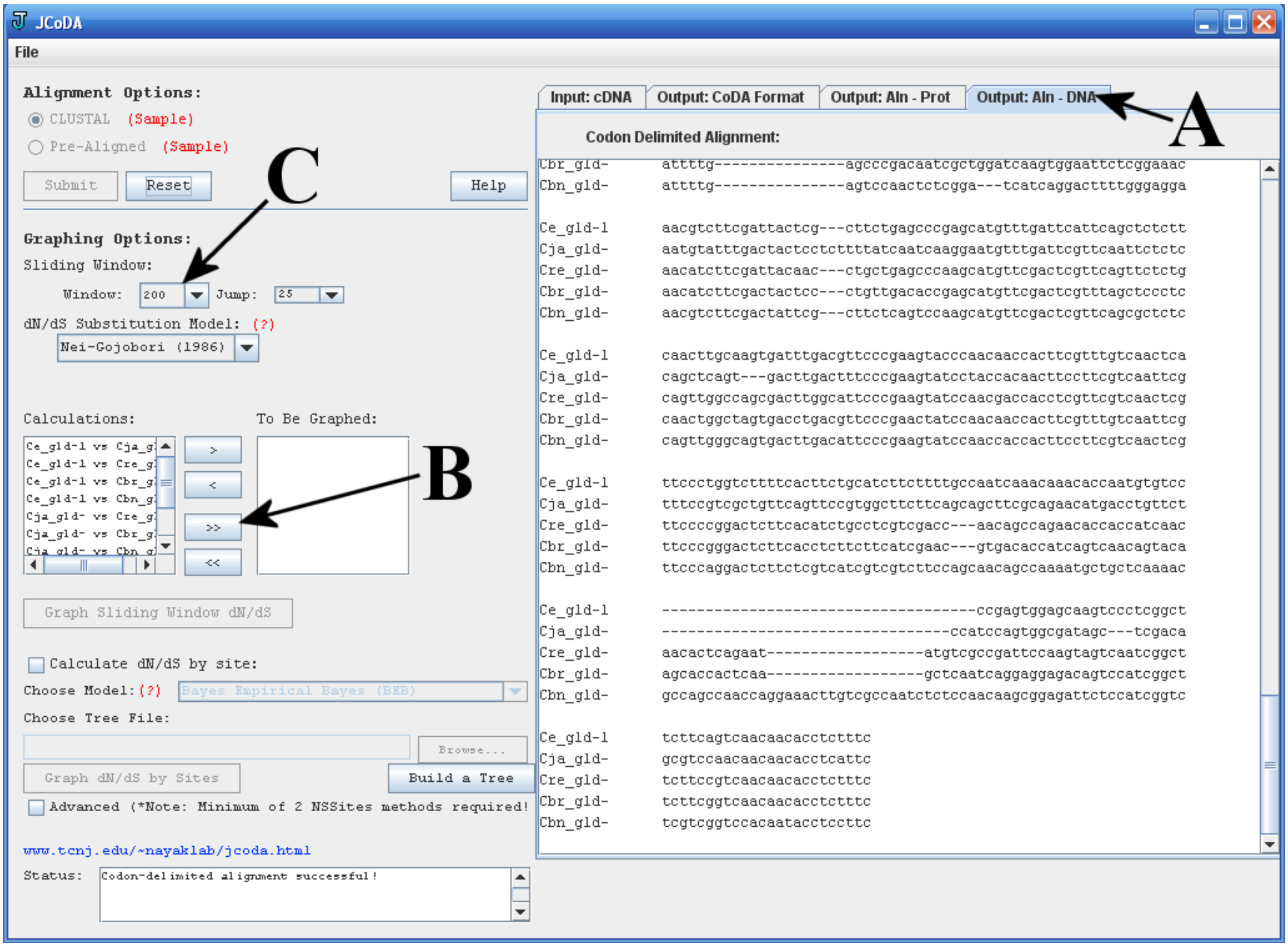

- Tabs allow for switching between sequence views. Codon-delimited alignment of the gld-1 CDS file is shown (A)
- Select sequences for comparison (B). Individual or all comparisons can be selected using the shuttle buttons. All comparisons were used for this analysis
- Specify window, jump, and model for analysis using the pull-down menus (C). Window of 100 and jump of 10 were used for this analysis

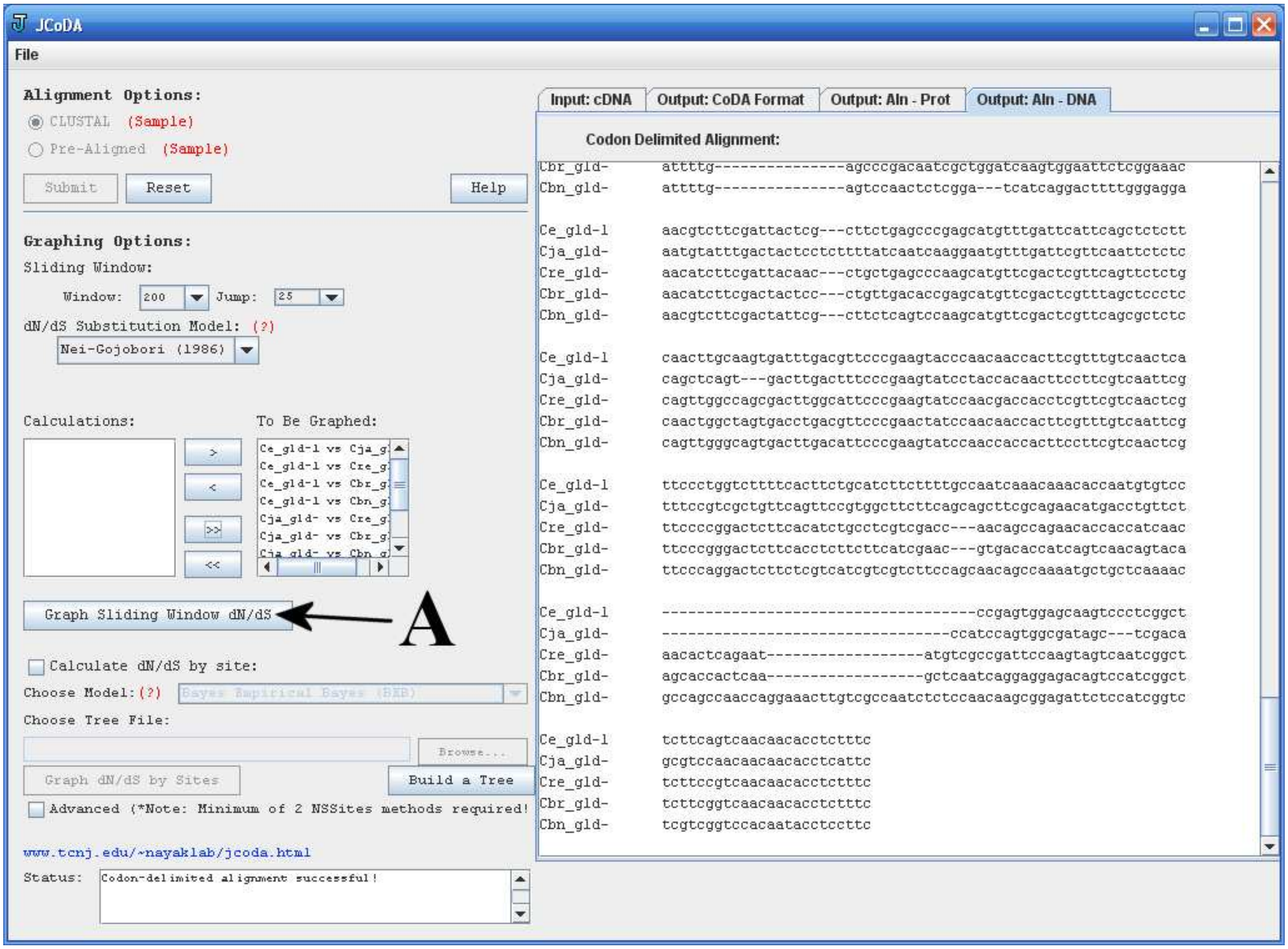

- After desired comparisons and parameters have been specified click "Graph Sliding Window dN/dS" (A)
- **Please Be Patient!** Depending on the number of comparison selected, graphing parameters, and the speed of your machine this can take some time

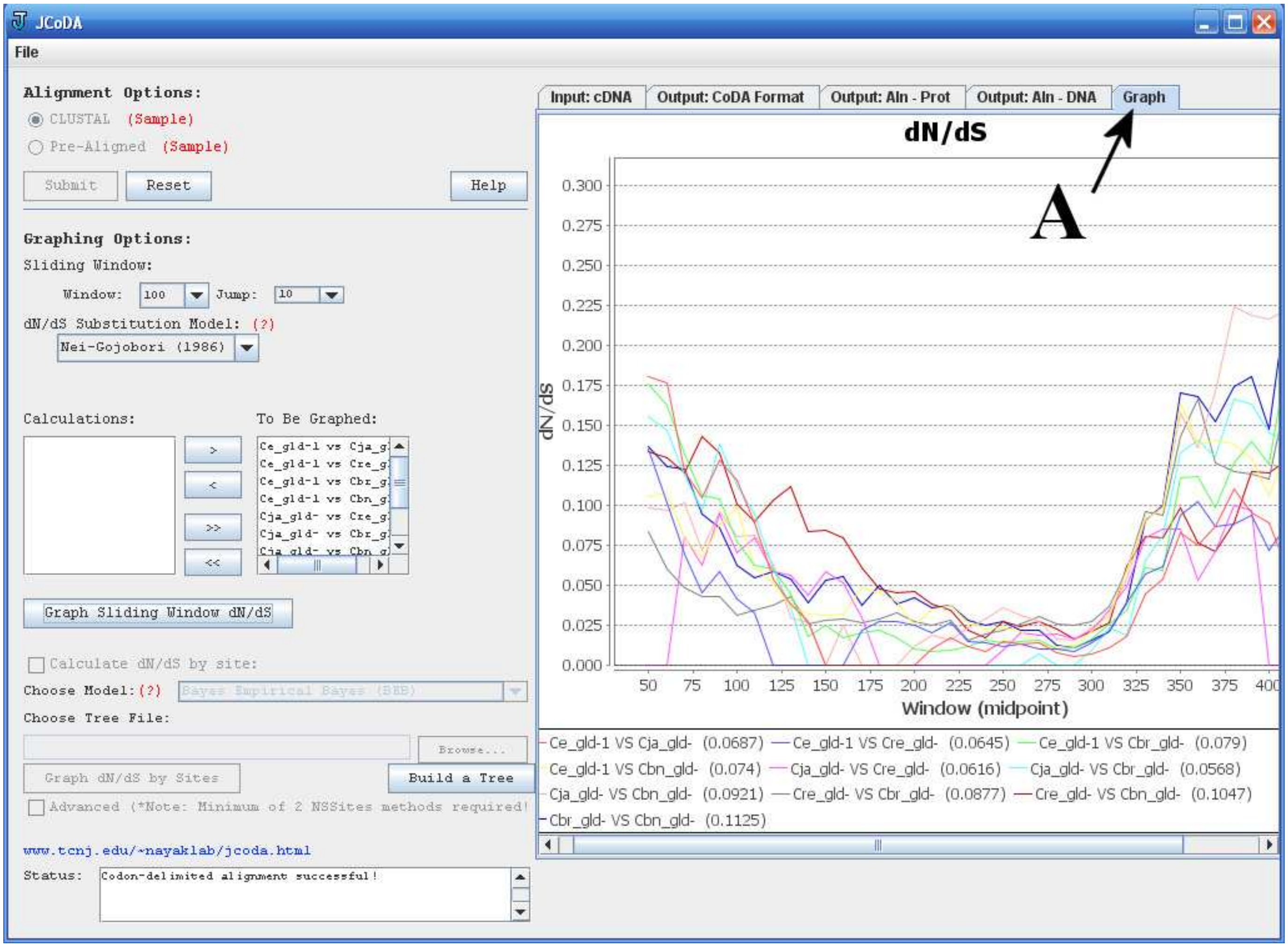

- The dN/dS sliding window graph can be viewed by clicking the "Graph" tab (A)
- Graphs generated with alternative parameters (e.g. bigger/smaller windows, different substitution models, different sequences selected) for comparison will appear as additional tabs to the left of the original "Graph" tab for comparison

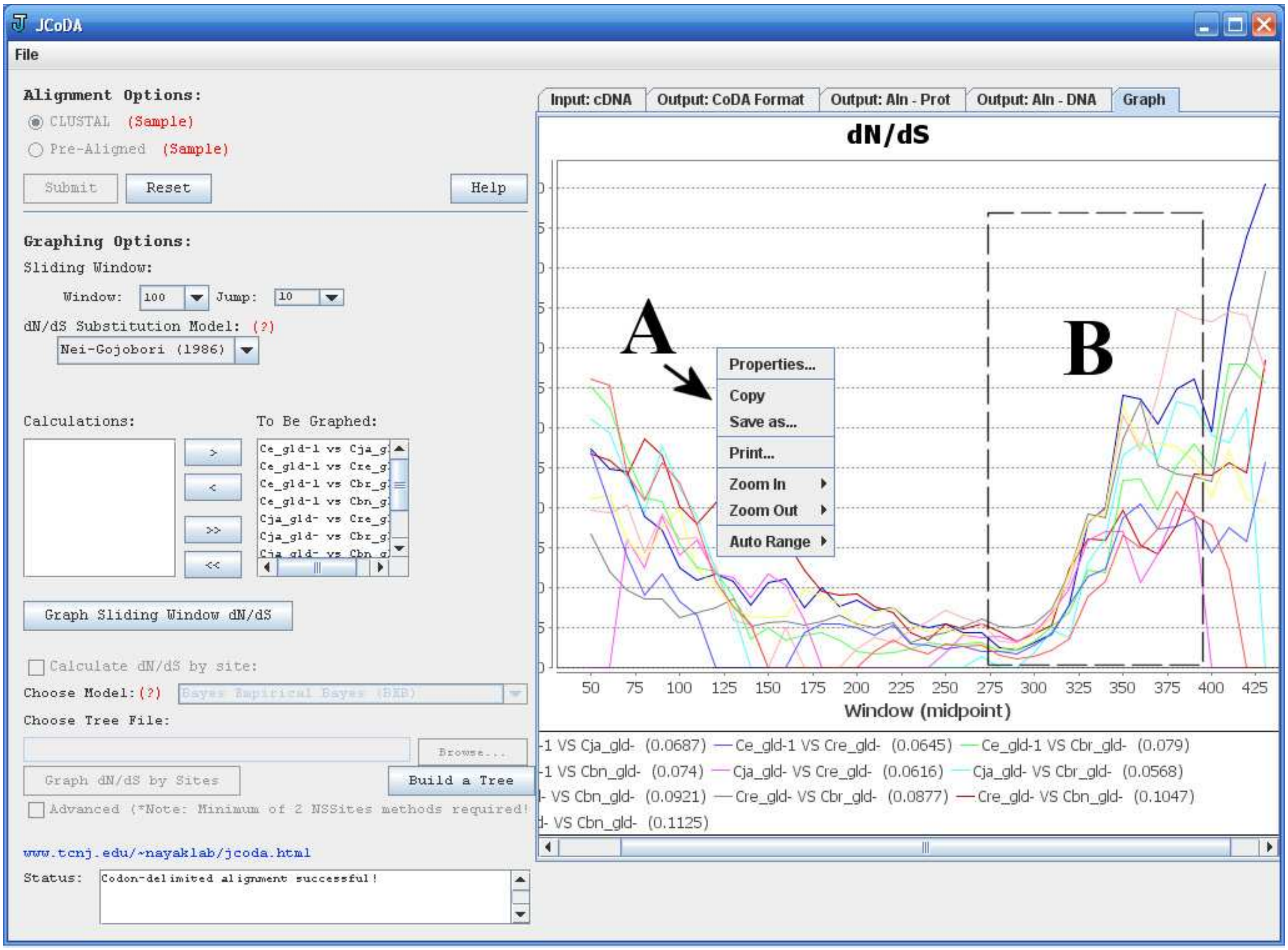

- Right-click anywhere on the graph to access graph properties and save options (A)
- The graph can be dynamically scaled using left-click and selecting the area desired. The area selected for this analysis is indicated by the dashed box (B, see below)

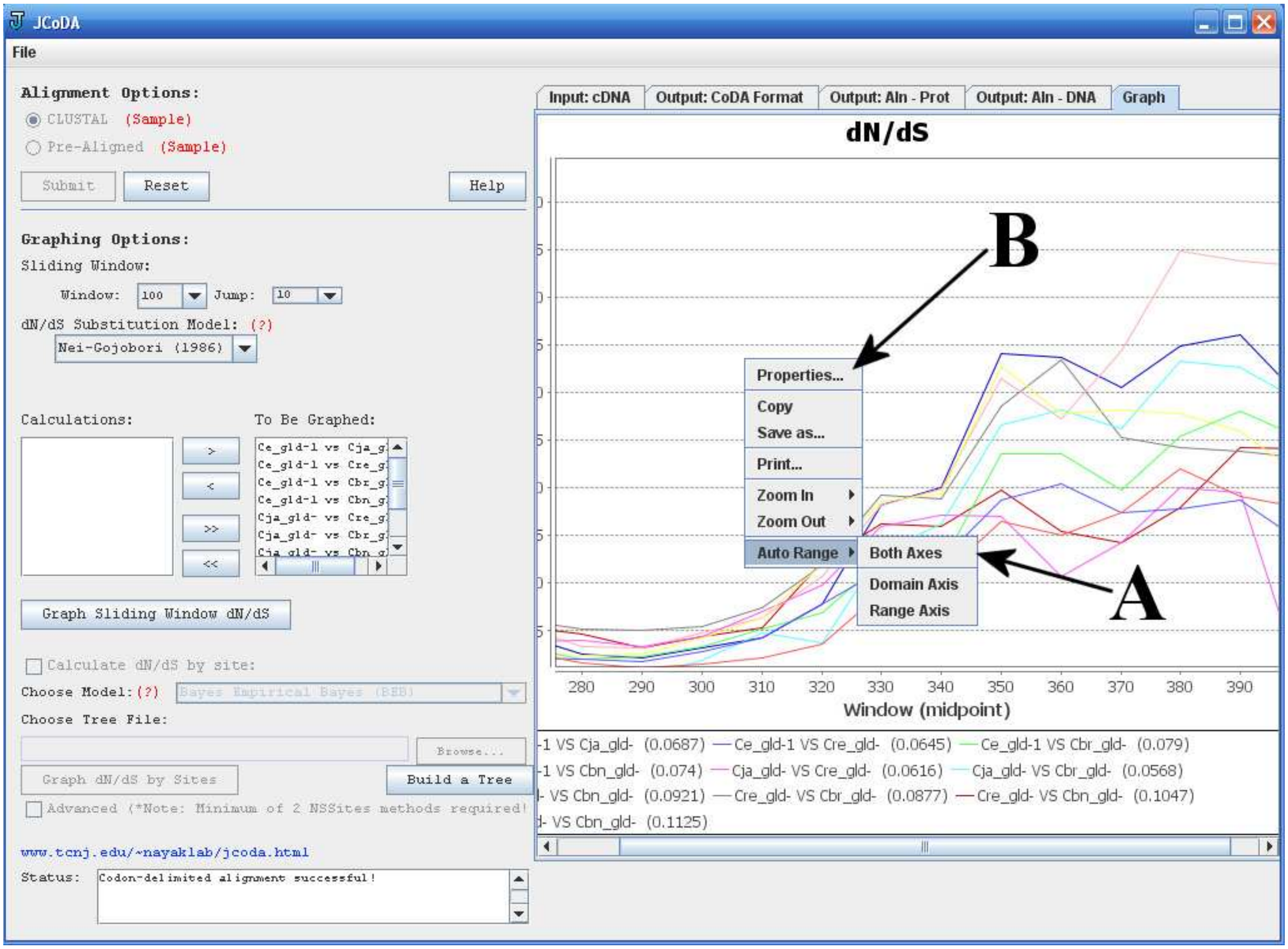

- To return to the main graph right-click anywhere on the graph and select "Auto Range" and "Both Axes" in the submenu (A)
- To change graph properties such as title, axes, fonts, lines, and other common parameters you can right-click anywhere on the graph and select "Properties" (B)
- Once you have modified the parameters to your liking remember to save it in its final form by right-clicking anywhere on the graph and selecting "Save as"
- If you have generated multiple graphs you must save each graph you wish to keep individually

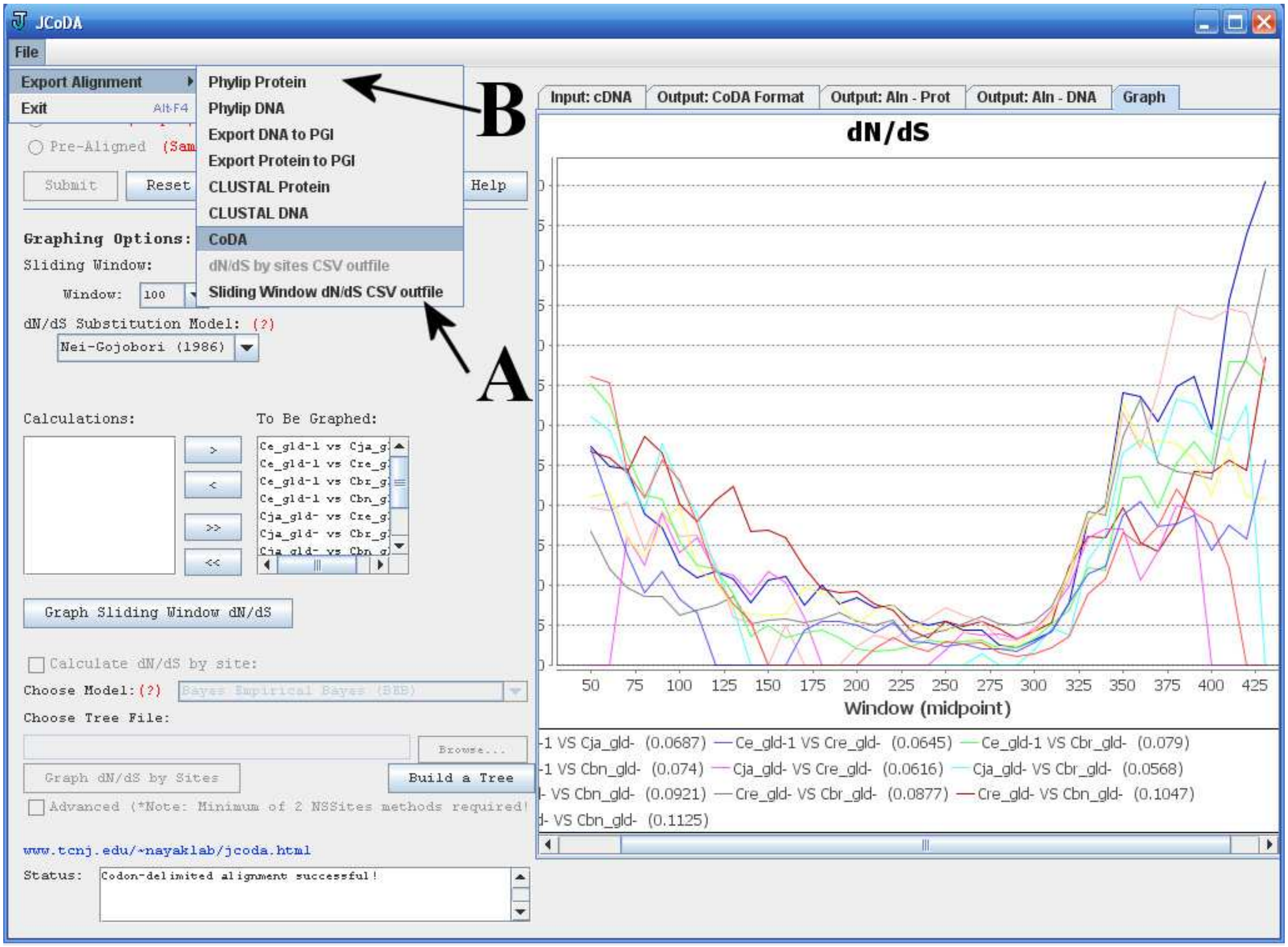

- All data from sliding window based dN/dS analysis can be saved as a single CSV file for down stream analysis (A)
- From the File menu -> select Export Alignment -> select Sliding Window dN/dS CSV outfile
- The exported file can be imported directly into programs such as Microsoft Excel that support CSV format
- If you are planning on doing dN/dS (**Section 3.1**) by site and/or need a phylogenetic tree then export the alignment in Phylip format (B)

3.1 dN/dS calculation using site based methods

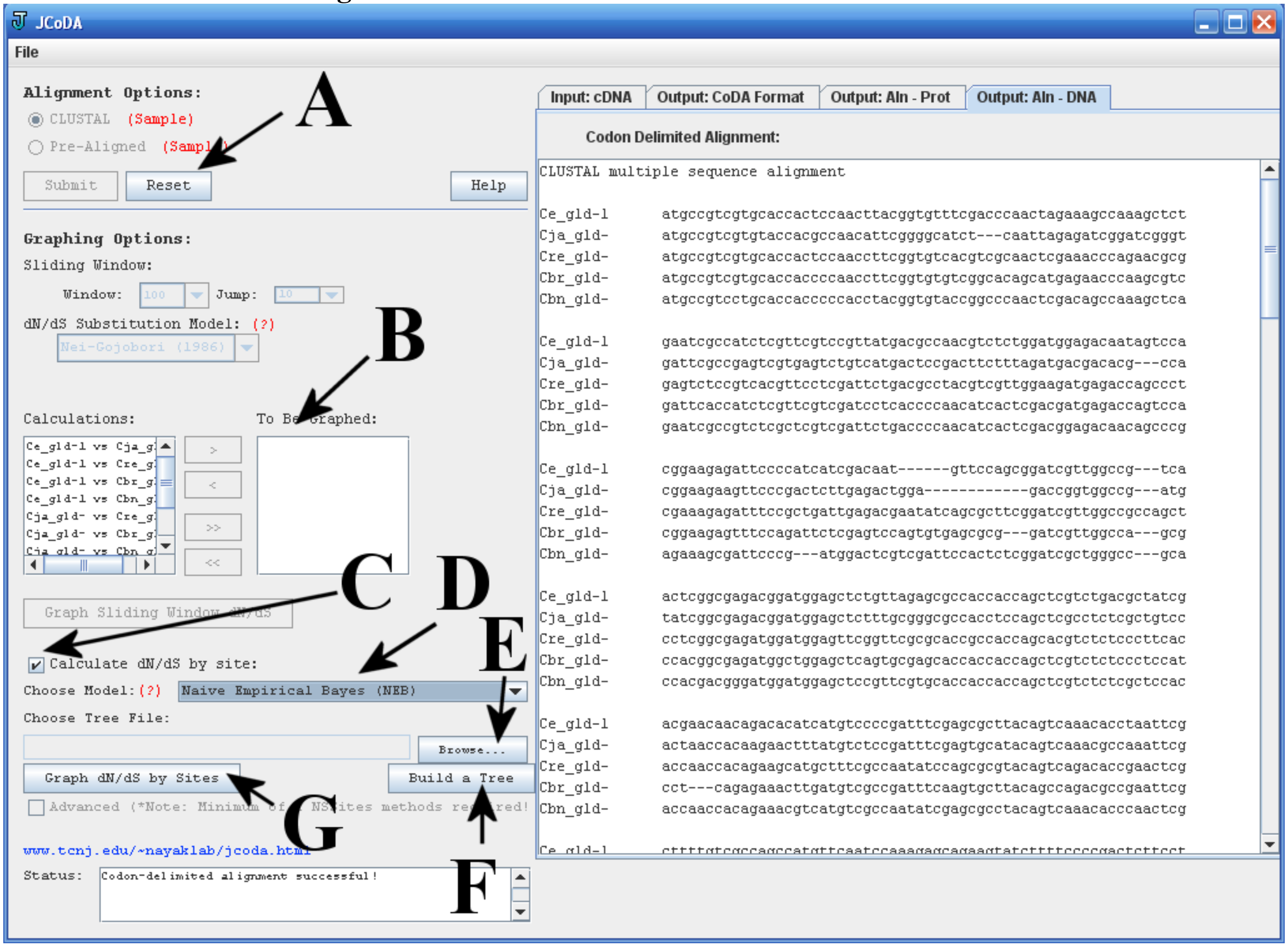

- Begin by pressing the Reset button if you have been using JCoDA for other analysis (A)
- <u>*See section 2.1 to generate codon-delimited alignment before starting.*</u> Gld-1 CDS from sample data are shown
- Once the alignments have been processed, select the sequences to be compared (B). NOTE: The sequences must match the sequences used for the tree in part E (see below)
- Select "Calculate dN/dS by site" (C)
- Choose a model (D). BEB is shown below
- This analysis requires a tree file in Phylip format. Point JCoDA to the tree file using the "Browse" button (E). A GLD-1 tree file has been provided in sample data for use with this analysis. If you are using your own tree file make sure that the names in the alignment match the names in the tree file exactly (see Troubleshooting and FAQ section if you have problems)
- If you do not have a tree file click the "Build a Tree" button and see **section 4.1** to generate a tree using PGI (Phylip Graphical Interface) (F)
- Regardless of the source of the tree file the names in the tree file and the names (see FAQ)
- Click "Graph dN/dS by Sites" once the path for the tree file has been specified (G)
- **PLEASE BE PATIENT!** It is unlikely that JCoDA has crashed. Depending on the number of species involved and the speed of your machine this analysis can take a considerable amount of time. If you are running the analysis through a virtual machine it can take even longer

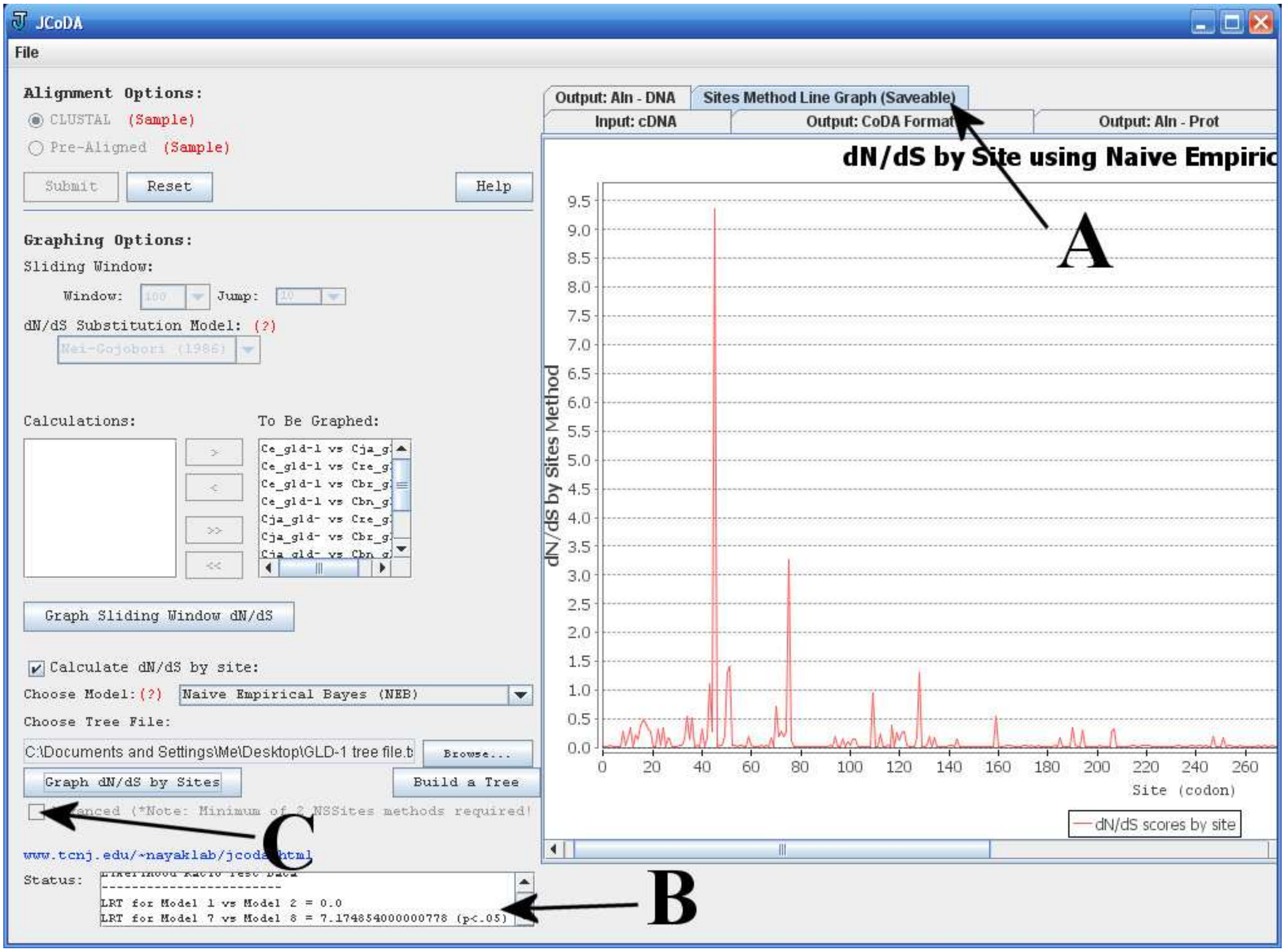

- Graph of dN/dS by site graph can be viewed by on the tab (A)
- Likelihood ratio test (LRT) defaults to Model 1 –vs- Model 2 and Model 7 –vs- Model 8. Evidence for positive selection under each model comparison is indicated by $p < 0.05$
- If you run additional analysis with new parameters the graph will appear as a tab to the left of the original
- The "Advanced" button provides access to the codeml control file were other options can be varied (For example, additional models can be specified and substitution models can be changed). *See Section 3.2 before using!*

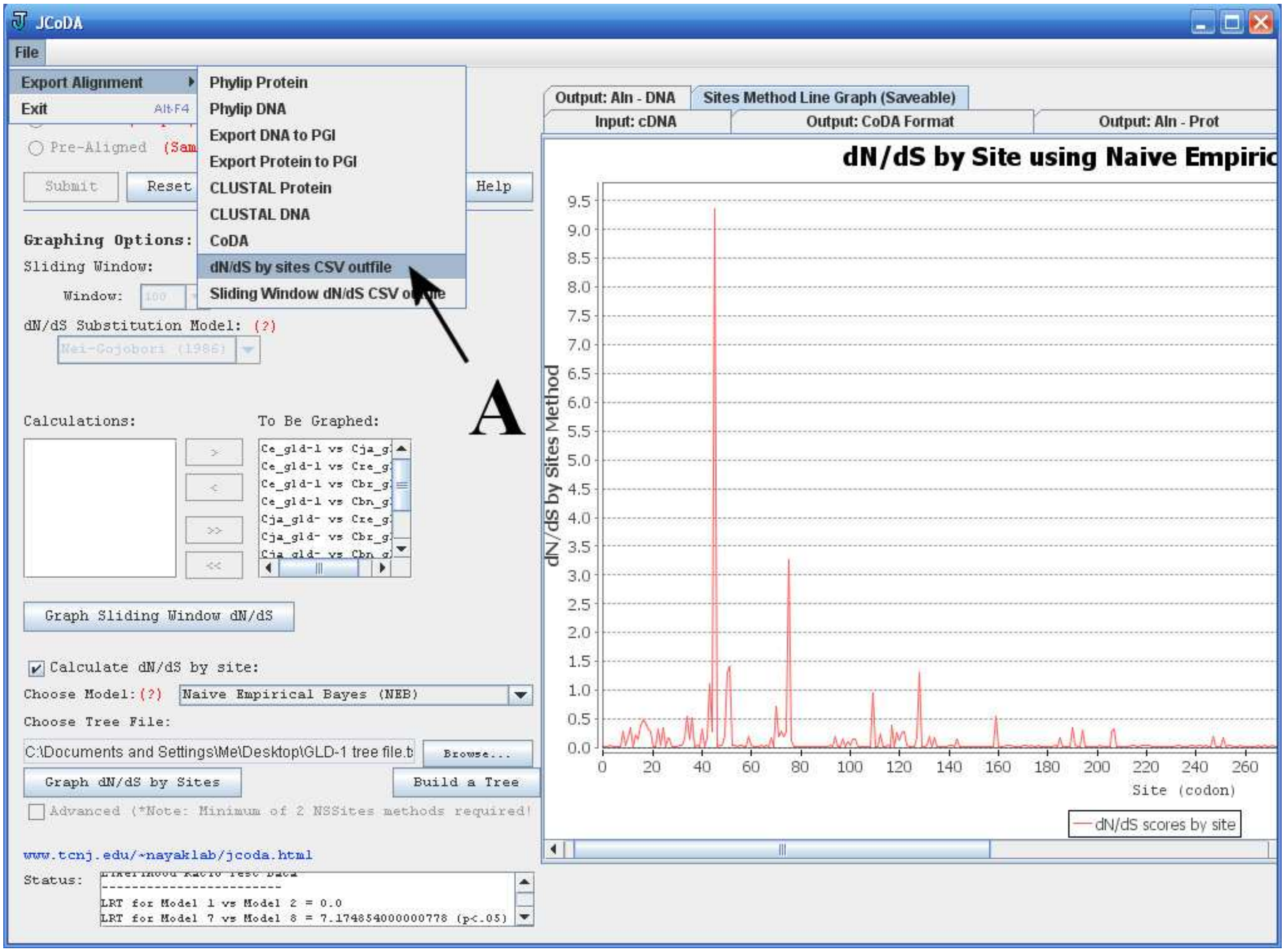

- All data from site based dN/dS analysis can be saved as a single CSV file for downstream analysis
- From the File menu -> select Export Alignment -> dN/dS by sites CSV outfile (A)
- The exported file can be imported directly into programs such as Microsoft Excel that support CSV format

3.2 dN/dS advanced options

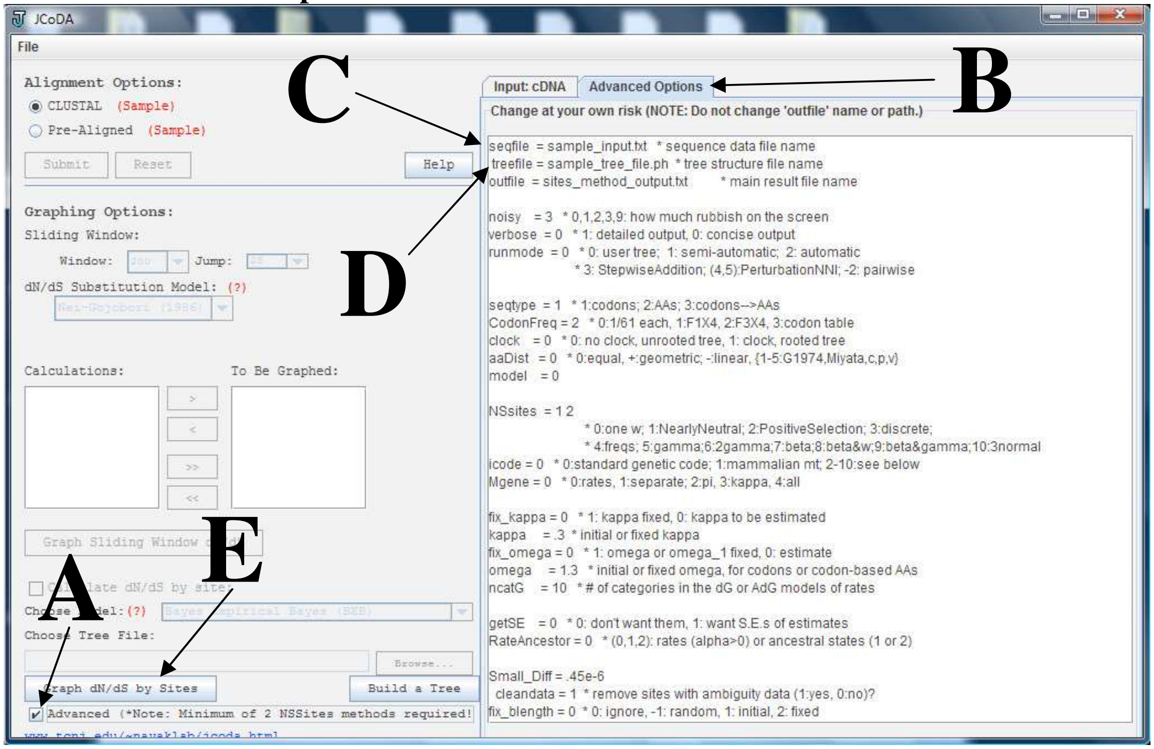

- This option is intended to mimic PAML. To use advanced options launch JCoDA and click on the “Advanced” check box (A) and the codeml control file will appear in a tab (B)
- The PAML control file requires a seqfile (C) and a treefile (D)
- If you have JCoDA on your desktop then these files are can be found by navigating as follows: Desktop->JCoDA_distribution->paml->advanced_options
  PATH: Desktop\JCoDA_distribution\paml\advanced_options
- The seqfile contains the alignment you want to use in Phylip format. The file to illustrate functionality, the current seqfile is called “sample_input.txt” (the txt extension may be hidden) and contains a small subset of TGFβ sequences. You can either replace these sequences with your sequences in the same Phylip format OR place a new file in the “advanced_options” folder and provide the name to the control file (C).
- The treefile contains the tree you want to use in Phylip format. The file to illustrate functionality is called “sample_tree_file.ph.txt” (the txt extension may be hidden) and contains a tree for use with the TGFβ sequences. You can either replace contents of the treefile with your tree in Phylip format OR place a new treefile in the “advanced_options” folder and provide the name to the control file (C).
- Once you have the sequences and tree file in place that you want – click on “Graph dN/dS by Sites”. PAML will use these files to perform the requested analysis with any parameters you have changed and JCoDA will retrieve and graph the results.
- All data associated with the analysis will be in the “rst” file and the “sites_method_output” file. If you want the information from these files then copy and paste them to the location you want. Do not change the names or paths of these files.
- **NOTE:** This option runs independently and does not automatically transfer sequences form previous analysis.

4.1 Generating trees using the Phylip Graphical Interface (PGI)

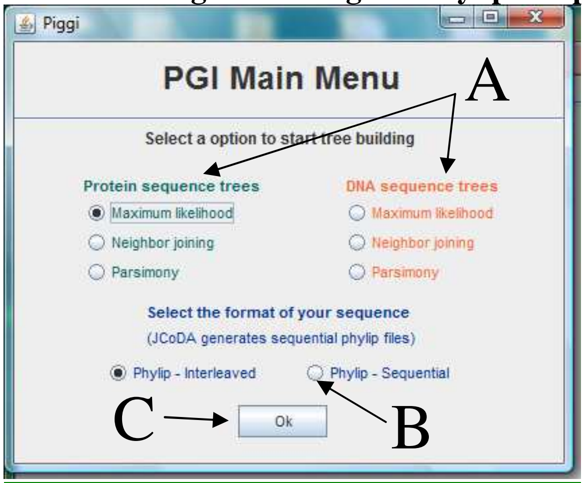

- Select the type of sequence (DNA or Protein) and the method (A). JCoDA defaults to maximum likelihood and this methods is implemented for the rest of this analysis
- Select the input format of the sequence (B). Select "Phylip - Sequential" to use with Phylip file generated in **Section 2.1**
- Click Ok (C)

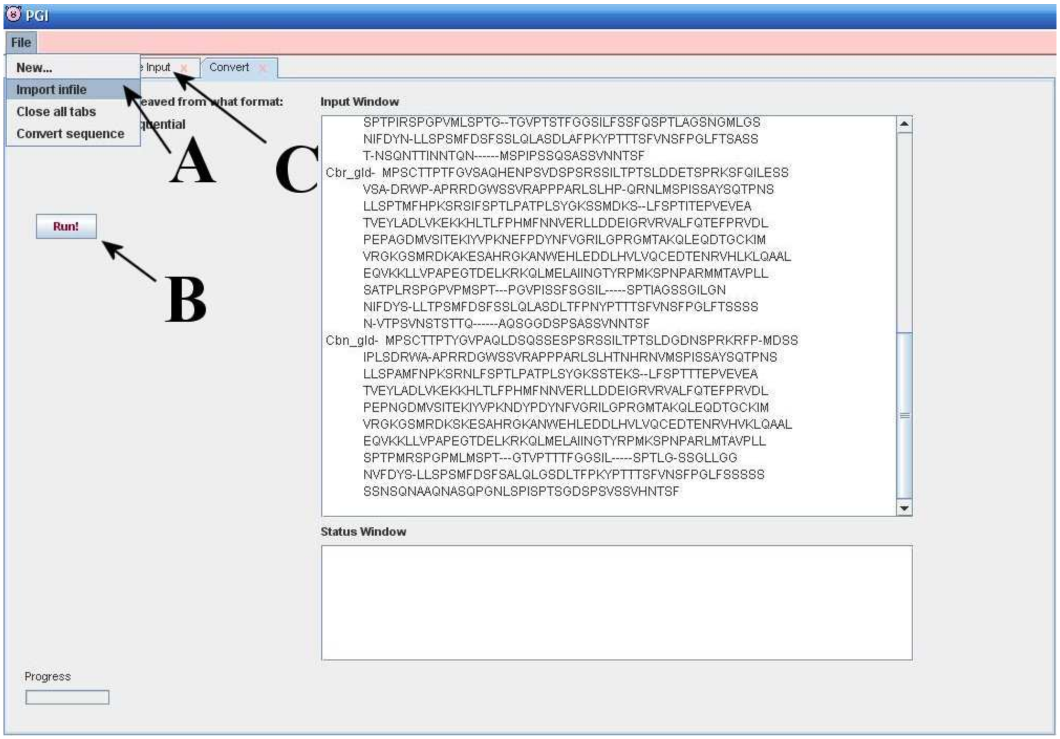

- From the File menu (A) select "Import infile" and direct the browser to the Phylip format file generated at the end of **Section 2.1**
- Click "Run!" (B) to convert the file for use with Phylip. If you already have a Phylip interleaved file you can skip this step. The converted file will be in the JCoDA main folder as "convertedFile"
- Click the Maximum Likelihood Tree Input tab to begin building the tree (C)

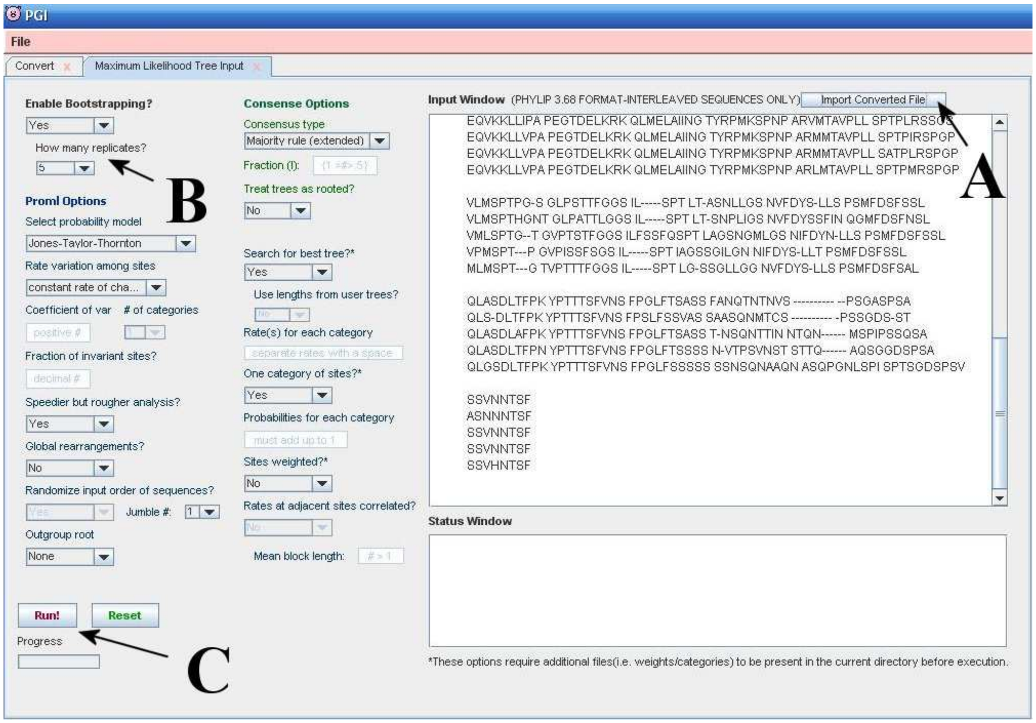

- Simply press the “import converted file” button located on top of the input window. The contents will appear in the Input Window (A)
- If you have your own infile in Phylip interleaved format you can import that directly (File -> Import infile)
- JCoDA defaults to using bootstrapping (can be turned off from first pull-down menu). For this example, 5 replicates were selected from the pull-down menu (B)
- Once you set your parameters click the Run button (C). This sample analysis uses the default parameters for all other parameters

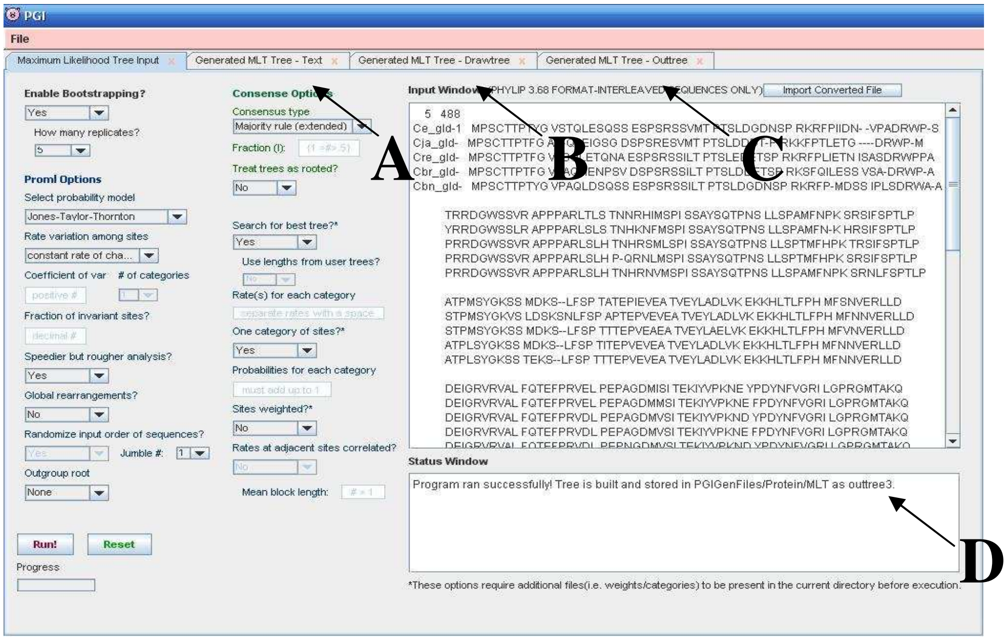

- Once the analysis is complete the text version of the tree (A), output from Drawtree (B), and tree file (C) are available as clickable tabs. The tree file is also saved in PGIGenFiles ->Protein -> MLT -> outtree3 (D). The exact path will vary based on your analysis but will always be in the PGIGenFiles folder

4.2 Exporting sequences from JCoDA to PGI

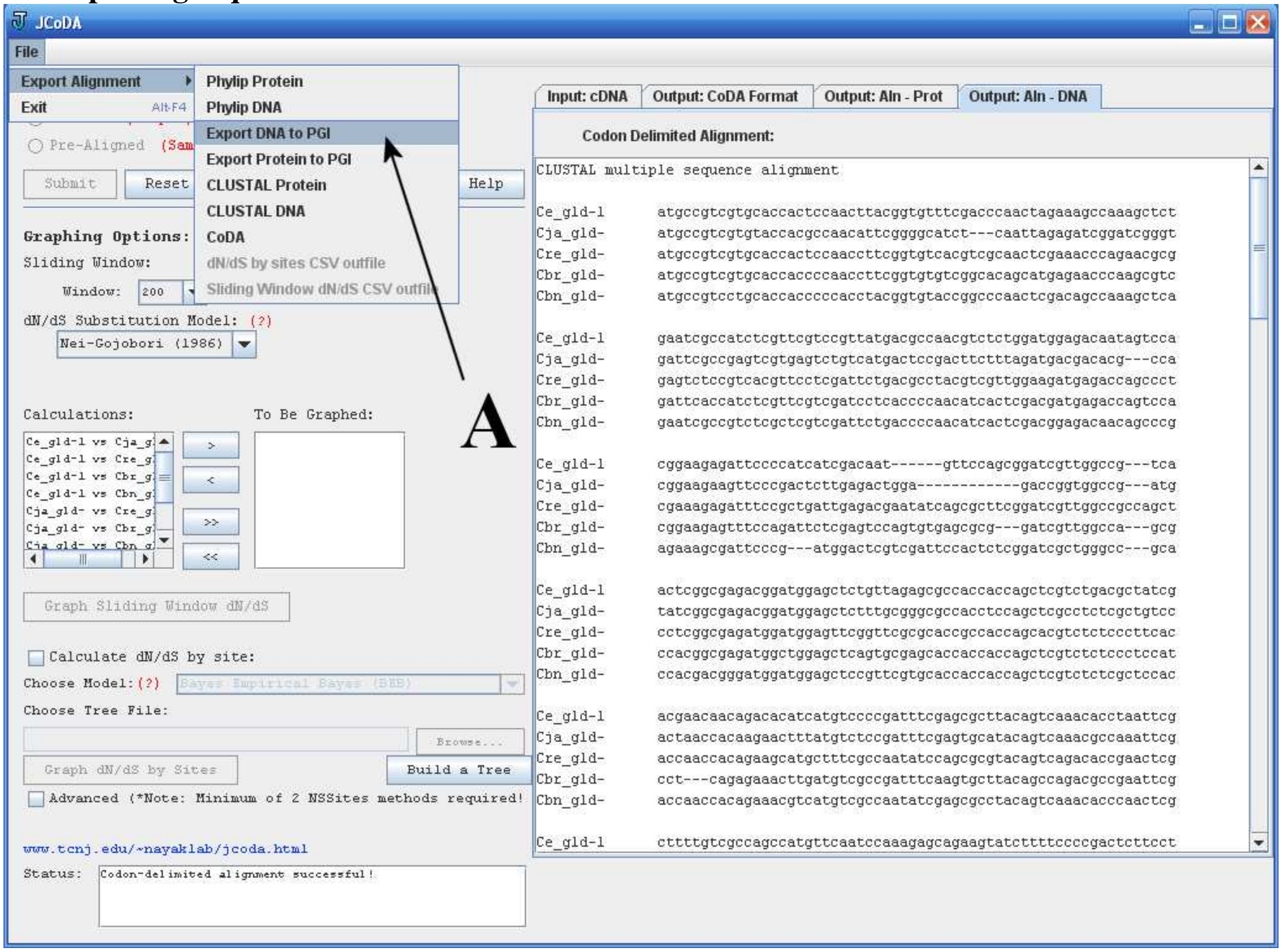

- After submitting a cDNA sequence, click File ->Export Alignment->Export DNA to PGI (A). An alert window will tell you that PGI will be opened and that you must convert the sequence, press ok.

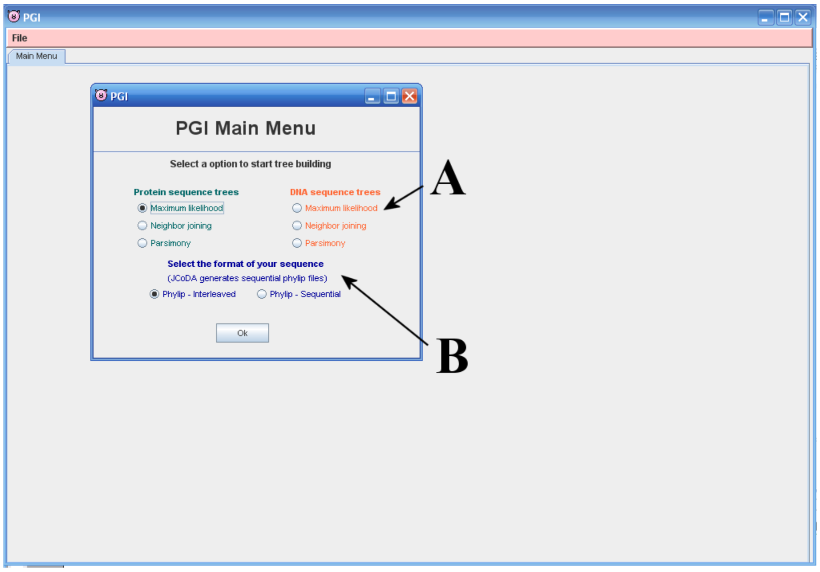

- When PGI is launched you will be presented with the main menu. Choose what you type of tree you want to build (A), and disregard the option in (B) (either option will bring up the convert tab) then press ok.

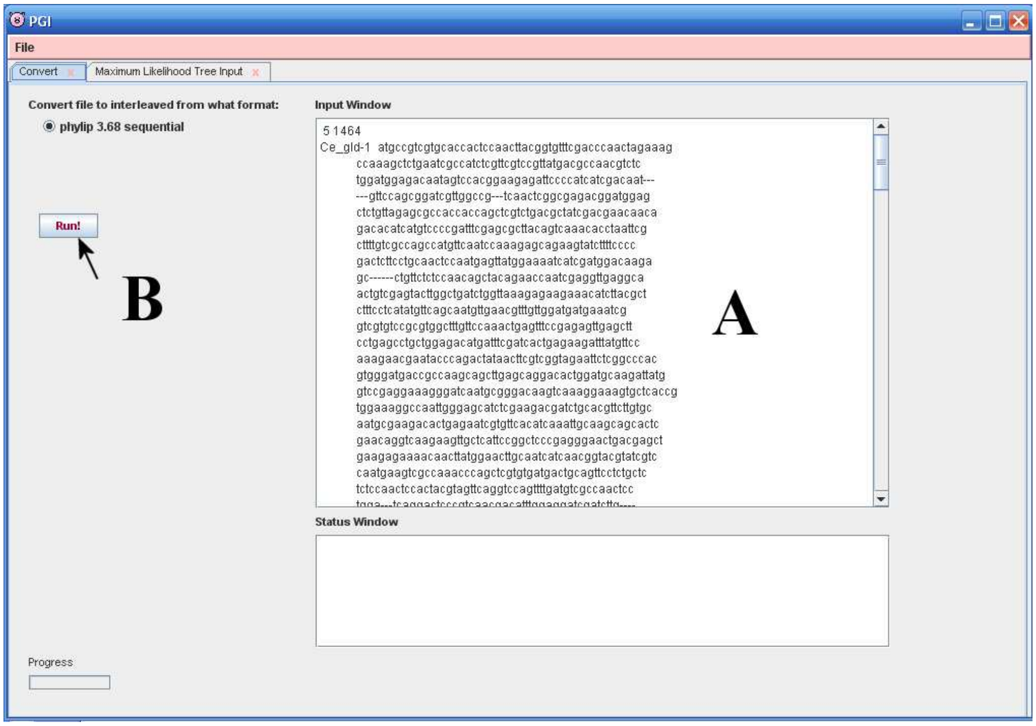

- Your sequence from JCoDA should be in the input window (A). Click “Run!” to convert (B).
- The converted sequence will be saved in the folder that contains the JCoDA executable and will be called convertedFile.

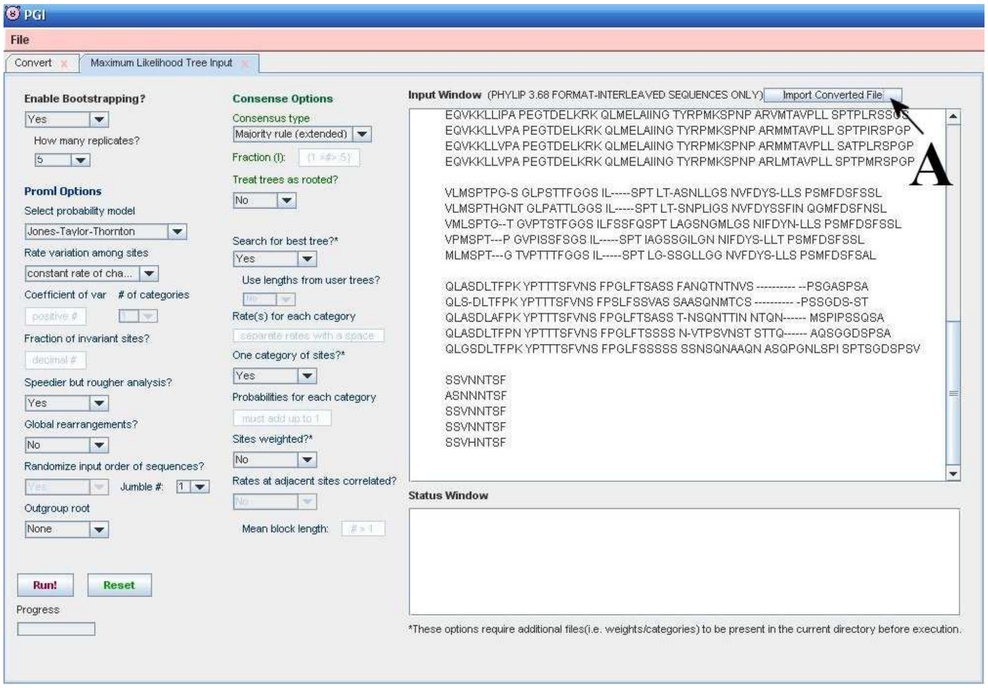

- Click on the “Import Converted File” button and the convertedFile will be put into the input window (A)
- Now continue using PGI normally.

5.1 Troubleshooting and FAQ

JCoDA will not start on my Windows PC! What's wrong with it?
*A video of this type of problem is in the docs/videos folder.
JCoDA requires Java Runtime Environment 6 (and Java 1.6) or Java Developer Kit 6 (includes Java 1.6 and Java Runtime Environment). Either can be freely downloaded at http://java.sun.com/javase/downloads/index.jsp. Even if you have Java installed on your Windows PC, JCoDA requires both Java 1.6 and Java Runtime Environment. Installation of Java Developer Kit 6 is recommended.

JCoDA will not start on my Mac running Boot Camp!
If you have Windows XP running on a separate partition make sure that correct versions of Java and JRE are installed. If JCoDA will not start or is crashing check the path in the environment variables. These issues are addressed in Section 1.2. If issues persist using JCoDA with Boot Camp (Windows XP) an alternative is to a virtual machine (see below).

JCoDA will not start on my OS X 10.5.x Mac even when I install Parallels (or VMware, or VirutalBox)! What's wrong with it?
If you have installed virtual machine software make sure you have a Windows virtual machine installed running XP, Vista, or 7 AND have installed Java Runtime Environment 6 (and Java 1.6) or Java Developer Kit 6.

When I click the JCoDA executable jar file nothing happens? What's wrong?
This can happen if you try to run JCoDA without extracting all the files from the downloaded archive. Unzip the JCoDA distribution and try running the executable again.

JCoDA is running but has limited functionality or is not working properly!

- **It's not performing a codon-delimited alignment.**
- **It only works some of the time.**
- **It's not allowing for sliding window analysis of pairwise dN/dS.**
- **It works with pre-aligned sequences but does not work with CDS that I paste in.**

*A video illustrating this type of problem is in the docs/videos folder.
JCoDA actually will run natively in OS X and other operating systems with very limited functionality. To resolve this issue, take the following steps: First, make sure that the VM has fully loaded, Windows (XP, Vista, or 7), and everything has finished updating. Second, double-click the JCoDA executable from inside the virtual machine for full functionality. For example, if you are in OS X and double click the JCoDA jar file it will be run under OS X. For JCoDA to function properly you must enter the VM first and then double click the JCoDA jar file.

I have a protein alignment that I modified by hand. How can I use my alignment with JCoDA?
Select Pre-aligned from Alignment Options section and paste in your protein sequence as aligned FASTA format in the top window and the corresponding CDS sequence in the window below.

I already have a Phylip tree file. Do I still have to use the Phylip Graphical Interface?
No. Any tree file in Phylip format will work.

I just want to use the Phylip Graphical Interface. Do I still have to launch JCoDA?
No. The Phylip Graphical Interface (PGI) can be run independently of JCoDA by clicking on "PGI" in the main directory. PGI can be used as a standalone tool for phylogenetic analysis.

The sliding window method worked but why is the site-based method is not doing anything. What's wrong with it?
There is a discrepancy between the names in the user provided tree file and the Phylip interleaved alignment file. Check the names in the tree file and the sequence file, make sure they are identical and that the first eight characters are unique.

I already have a Phylip tree file but the names don't match the sequence in the alignment file. How do I edit the tree file so the names match?
You can use any text editor to view and change the names in the tree file. As long you save the file as "text only" you can edit the file using Microsoft Word. If you are operating through a virtual machine on a Mac DO NOT use SimpleText – use Notepad (or Word, saving as text only) to avoid problems with line endings.

I made my tree file using another different phylogenetic inference program. How do I get my tree file to work with JCoDA?
JCoDA will accept any tree file in Phylip format provided that the names in the tree file and the names in the alignment file are identical. Edit your tree file so that the names are identical to the sequence file.

I have a Mac. Can I run JCoDA using Mac Parallels, VMware, VirtualBox or other virtual machine?
As of right now no compatible version has been written that is fully functional natively on OS X. JCoDA is fully functional on Macs using a virtual machine (Parallels, VMware, VirutalBox, etc.) provided you have also installed Windows XP, Vista, or 7 and Java Developer Kit 6.

I am using Linux, can I run JCoDA?
As of right now no compatible version has been written that runs directly on Linux. However, with some modifications to the source code and the ClustalW, PAML, and Phylip executables it is possible to run JCoDA on a Linux machine. JCoDA has not been tested on Linux using a Windows virtual machine.

I've noticed a few bugs, what can I do?
If you find any bugs or glitches let us know at nayak@tcnj.edu.

Is there a way to run JCoDA from the command line?
Yes. You can use Java's Jar command, simply navigate to the location of the executable jar file and enter the command "java -jar JCoDA.jar" and likewise for PGI "java –jar PGI.jar".

Can I modify JCoDA? I want to add functionality, streamline some of the processes, improve the code, change/improve the interface, add automation, etc.
Yes. You are free to modify JCoDA or PGI provided you do not violate the copyright or terms of use for ClustalW, Phylip, PAML, and any other programs or source code you implement.

6.1 References

1. Thompson, J. D.; Higgins, D. G.; Gibson, T. J., ClustalW: improving the sensitivity of progressive multiple sequence alignment through sequence weighting, position-specific gap penalties and weight matrix choice. *Nucleic Acids Res* **1994,** 22, (22), 4673-80.
2. Felsenstein, J., Phylip - Phylogeny Inference Package (Version 3.2). *Cladistics* **1989,** 5, 164-166.
3. Yang, Z., PAML 4: phylogenetic analysis by maximum likelihood. *Mol Biol Evol* **2007,** 24, (8), 1586-91.
