# Supplementary figures and images for: JCoDA: a tool for detecting evolutionary selection
Source: BMC Bioinformatics. 2010 May 27;11:284. doi: 10.1186/1471-2105-11-284 (PMC2887424; doi:10.1186/1471-2105-11-284)

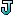

Supplement: Additional file 6 — Zipped archive that contains all JCoDA source code and executable jar files. [file 1471-2105-11-284-S6.ZIP › JCoDA/lib/JLogo.png]

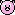

Supplement: Additional file 6 — Zipped archive that contains all JCoDA source code and executable jar files. [file 1471-2105-11-284-S6.ZIP › JCoDA/lib/PiggiLogo.png]

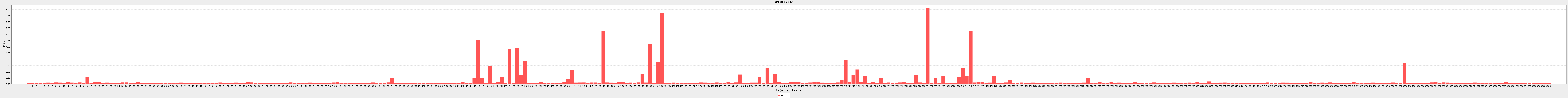

Supplement: Additional file 6 — Zipped archive that contains all JCoDA source code and executable jar files. [file 1471-2105-11-284-S6.ZIP › JCoDA/paml/advanced_options/sites_image.png]

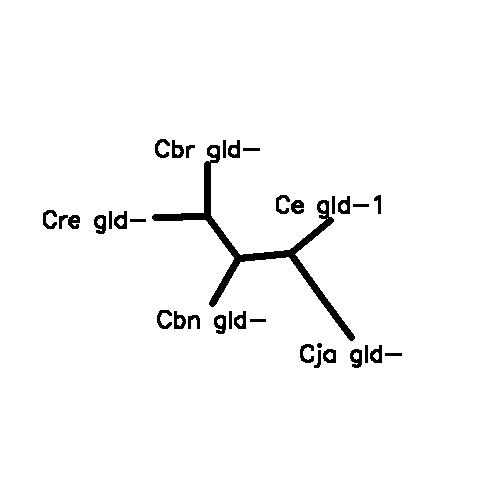

Supplement: Additional file 6 — Zipped archive that contains all JCoDA source code and executable jar files. [file 1471-2105-11-284-S6.ZIP › JCoDA/PGIGenFiles/Protein/MLT/plot.jpg]

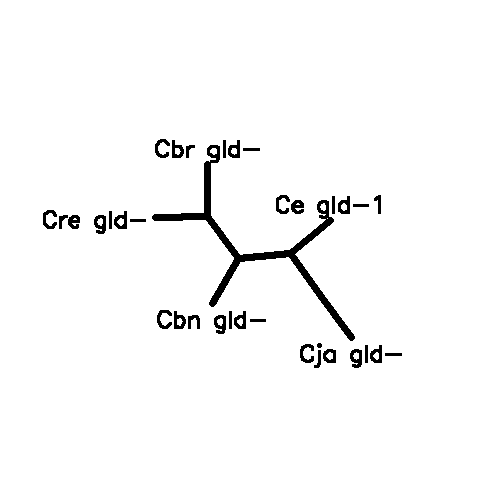

Supplement: Additional file 6 — Zipped archive that contains all JCoDA source code and executable jar files. [file 1471-2105-11-284-S6.ZIP › JCoDA/plotfile]
